# Aerosol inhalation of dimeric artesunate phospholipid-conjugated liposomes ameliorates inflammation, fibrosis, and ferroptosis in neonatal mice with hyperoxia-induced lung injury

**DOI:** 10.3389/fphar.2025.1542743

**Published:** 2025-07-21

**Authors:** Rong Guan, Yu Chen, Qianqian Yu, Bingrui Yu, Shuyu Chen, Siyuan Jia, Huifang Wang, Huaiping Cheng, Zhaofang Tian

**Affiliations:** ^1^ Department of Neonatology, The Affiliated Suqian First People’s Hospital of Nanjing Medical University, Suqian, Jiangsu, China; ^2^ Department of Neonatology, The Affiliated Huaian No. 1 People’s Hospital of Nanjing Medical University, Huai’an, Jiangsu, China

**Keywords:** bronchopulmonary dysplasia, hyperoxia, inflammation, fibrosis, ferroptosis, macrophages

## Abstract

Bronchopulmonary dysplasia (BPD), a chronic lung condition that impacts preterm infants, results in persistent lung damage with limited therapeutic interventions available. Artemisinin, a bioactive compound derived from Artemisia annua, a member of the Asteraceae family, exhibits potent anti-inflammatory and anti-fibrotic characteristics and has been proven to confer protective benefits against acute lung injuries triggered by various factors. However, its potential impact on BPD and the mechanisms involved are not fully understood. This research examines the function and fundamental processes of dimeric artesunate phospholipid-conjugated liposomes (Di-ART-GPC) in BPD. In the *in vivo* experiments, 48 male neonatal C57BL/6 mice were arbitrarily divided into four cohorts: air (NC cohort), air + Di-ART-GPC (NA cohort), hyperoxia (HO cohort), and hyperoxia + Di-ART-GPC (HA cohort). Mice in the NC and NA cohorts were exposed to normoxic conditions (21% O_2_) from birth, while those in the HO and HA cohorts were subjected to hyperoxic conditions (95% O_2_) for 7 days. On the eighth day, NC and NA mice were administered double-distilled water (ddH_2_O 4 mL), while HO and HA mice received Di-ART-GPC (0.5 mg dissolved in 4 mL ddH_2_O) *via* inhalation once daily for 3 days. Lung tissues and serum were harvested on postnatal day 11. Histological evaluations included HE staining for alveolar structure assessment and RAC count and inflammation score quantification; Masson staining for fibrosis evaluation; immunohistochemistry and real-time quantitative PCR (RT-qPCR) for detecting TGF-β1 and α-SMA expression; and ELISA for measuring TNF-α and IL-6 levels. Additional assays quantified superoxide dismutase (SOD), malondialdehyde (MDA), and glutathione (GSH) levels, while immunofluorescence and RT-qPCR assessed Gpx4 expression. For the *in vitro* component, RAW264.7 macrophages were categorized into the same four cohorts based on culture conditions. Cells in the NC and NA cohorts were cultured under normoxic conditions, while those in the HO and HA cohorts were exposed to 95% O_2_ for 24 h, following treatment with Di-ART-GPC at 1.25 µM. The supernatant and cells were harvested for subsequent examination. ELISA was employed to measure TNF-α, IL-6, and TGF-β1 levels in the supernatant, while Western blot and RT-qPCR were employed to assess Gpx4 expression in RAW264.7 cells. *In vivo* findings demonstrated that, in contrast to the NC cohort, the HO cohort exhibited disrupted alveolar architecture, widened alveolar spaces, reduced RAC values, and elevated inflammation and fibrosis scores (p < 0.05). Additionally, the HO cohort demonstrated elevated levels of IL-6 and TNF-α (p < 0.05), higher mRNA expression of TGF-β1 and α-SMA (p < 0.05), reduced SOD activity, diminished GSH content (p < 0.05), and diminished GPX4 protein expression (p < 0.05). Administration of Di-ART-GPC markedly improved these parameters (all p < 0.05). Similarly, *in vitro* experiments revealed that Di-ART-GPC treatment reduced IL-6, TNF-α, and TGF-β1 levels in hyperoxia-exposed RAW264.7 cells (p < 0.05) and enhanced GPX4 expression (p < 0.05). These findings indicate that Di-ART-GPC demonstrates safeguarding properties against hyperoxia-induced lung damage, potentially by mitigating inflammation and fibrosis in lung tissues and reducing macrophage ferroptosis in hyperoxia-induced BPD.

## 1 Introduction

Bronchopulmonary dysplasia (BPD) is a prevalent complication among preterm infants, particularly in those born extremely or very prematurely. It is one of the most significant and enduring chronic conditions associated with prematurity (Bell et al., 2022). Infants diagnosed with BPD face elevated mortality rates in early childhood and are prone to long-term health issues, including repeated hospitalizations, neurodevelopmental delays, and severe chronic lung disease. Consequently, BPD is increasingly recognized as a lifelong condition, extending far beyond the neonatal period and impacting long-term health outcomes (Bell et al., 2022; Stoll et al., 2015). Current clinical interventions primarily focus on symptomatic management, such as respiratory support and glucocorticoid administration. However, these treatments are linked to a heightened risk of complications, encompassing retinopathy of prematurity (ROP) and cerebral palsy. This underscores the critical need for advancing neonatal respiratory care and identifying safe, effective therapies for BPD (Principi et al., 2018; Michael et al., 2018). In this context, there is an urgent demand for further exploration of the underlying pathophysiological mechanisms of BPD and the development of novel therapeutic strategies (Martin et al., 2021).

BPD arises from multiple factors, including pulmonary immaturity, infections, nutritional deficits, oxygen toxicity, mechanical ventilation-induced injury, and inflammatory responses. Among these, exposure to hyperoxia remains the most prominent risk factor in preterm infants (Yang et al., 2023). Hyperoxia induces oxidative stress, characterized by pulmonary edema, inflammation, fibrin deposition, reduced surfactant activity, and the generation of highly reactive oxygen species (ROS) (Kimble et al., 2022). The subsequent oxidative stress and elevated ROS levels initiate iron-dependent lipid peroxidation, ultimately leading to cellular damage and death (Wen et al., 2024). Ferroptosis, an iron-reliant programmed cell demise marked by lipid oxidation (Chen et al., 2023), has recently been implicated in the pathogenesis of BPD (Chen et al., 2023). Research demonstrates that hyperoxia induces characteristic ferroptotic mitochondrial abnormalities in alveolar type II epithelial cells, and hyperoxia-exposed neonatal mice exhibit markedly decreased levels of glutathione peroxidase 4 (GPX4) and glutathione (GSH) in lung tissue (Jia et al., 2021; Chou and Chen, 2022a). These findings provide direct evidence that hyperoxia induces ferroptosis and impairs lung development in neonatal mice. Recent studies have revealed that ETS1 can ameliorate hyperoxia-induced BPD in mice by regulating Nrf2/HO-1-mediated ferroptosis (Yang et al., 2023). Thus, targeting ferroptosis represents a promising therapeutic approach for BPD management.

Artemisinin, an active compound derived from Artemisia annua of the Asteraceae family, was initially utilized primarily for malaria treatment (Shi et al., 2022). However, recent research has highlighted its diverse biological activities, encompassing anti-tumor (Ruwizhi et al., 2022), anti-fibrosis (Dolivo et al., 2021), anti-inflammatory (Efferth and OESCH, 2021), antiviral (Efferth, 2018), and immunomodulatory properties (Shi et al., 2022). Artemisinin and its derivatives usually have short half-life and poor bioavailability (∼30%) (Ismail et al., 2018). DHA, as the first-generation derivative of artemisinin, exhibits significantly enhanced antimalarial activity (Tu, 2016). However, its clinical application is limited by poor aqueous solubility and low bioavailability. To address these limitations, artesunate (ART) was subsequently developed from DHA with markedly improved water solubility. ART demonstrates complete dissolution in weakly alkaline solutions and is transported *in vivo* via passive diffusion, facilitating efficient biomembrane penetration. ART is primarily administered orally, characterized by rapid onset of action but suffers from a short *in vivo* half-life (approximately 1 h) (Benakis et al., 1997).

Dimeric artesunate phospholipid-conjugated liposomes (Di-ART-GPC), a novel derivative synthesized through the esterification of artesunate and choline glycerophosphate, exhibits low cytotoxicity with enhanced anti-inflammatory and antimalarial effects compared to ART alone (Ismail et al., 2018; Zhang et al., 2020). *In vitro* drug release and degradation results showed that the Di-ART-GPC were stable in neutral physiological conditions but effectively degraded to release parent ART in the simulated weakly acidic microenvironment; *in vivo* pharmacokinetics study revealed that Di-ART-GPC has a longer retention half-life in the bloodstream (Ismail et al., 2018). Recent studies have found that ART ameliorates chronic hyperoxia-induced BPD in neonatal mice by inhibiting the expression of NF-κB pathway and inflammatory factors (Weng et al., 2024). Furthermore, studies have revealed that artemisinin can attenuate liver fibrosis (Kong et al., 2019) and radiation-induced lung injury (Ning et al., 2024) through the regulation of ferroptosis. However, a notable lacuna exists in current research concerning whether artemisinin can mitigate BPD by targeting ferroptosis.

Aerosol inhalation, a non-invasive drug delivery method, offers several advantages, including increased local drug concentration in the lung, superior bioavailability, reduced systemic toxicity, and ease of administration (Elkasabgy et al., 2020). These characteristics make it particularly suitable for treating pulmonary diseases, especially pediatric and neonatal patients. In this study, aerosolized Di-ART-GPC was administered to hyperoxia-induced BPD mice to investigate its role and underlying mechanisms in alleviating lung injury. Additionally, *in vitro* experiments were conducted using hyperoxia-exposed RAW264.7 cells treated with Di-ART-GPC to assess its effects on macrophage activity and expression.

## 2 Methodologies and materials

### 2.1 Chemicals and reagents

Di-ART-GPC (purity ≥98%) was generously supplied by the Xinsong’s laboratory at Southeast University. RPMI Medium 1640 basic medium was obtained from Gibco (NY, United States), while kits for RT-qPCR, ELISA, SOD, MDA, and GSH were sourced from Proteintech (Wuhan, China). Antibodies for α-SMA, TGF-β1, GPX4, and ACTIN were purchased from Servicebio (Wuhan, China). Additional reagents, including RIPA Lysis Buffer, protease inhibitors, BCA Protein Assay Kit, and enhanced chemiluminescence exposure solution, were supplied by NCM Biotech (Suzhou, China). TRIzol reagent was procured from Invitrogen (CA, United States).

### 2.2 Animal model and intervention

C57BL/6J mice (25–30 g, 8–12 weeks old) were procured from Hangzhou Ziyuan Laboratory Animal Science and Technology Co., Ltd. (Hangzhou, China) and housed under SPF-grade conditions. Pregnant C57BL/6J mice were individually housed, and after natural delivery, male neonates less than 24 h old were selected for the experiment. All procedures were sanctioned by the Ethics Committee of the Affiliated Huaian No. 1 People’s Hospital of Nanjing Medical University (approval number: DW-P-2024-009-01).

**FIGURE 1:**
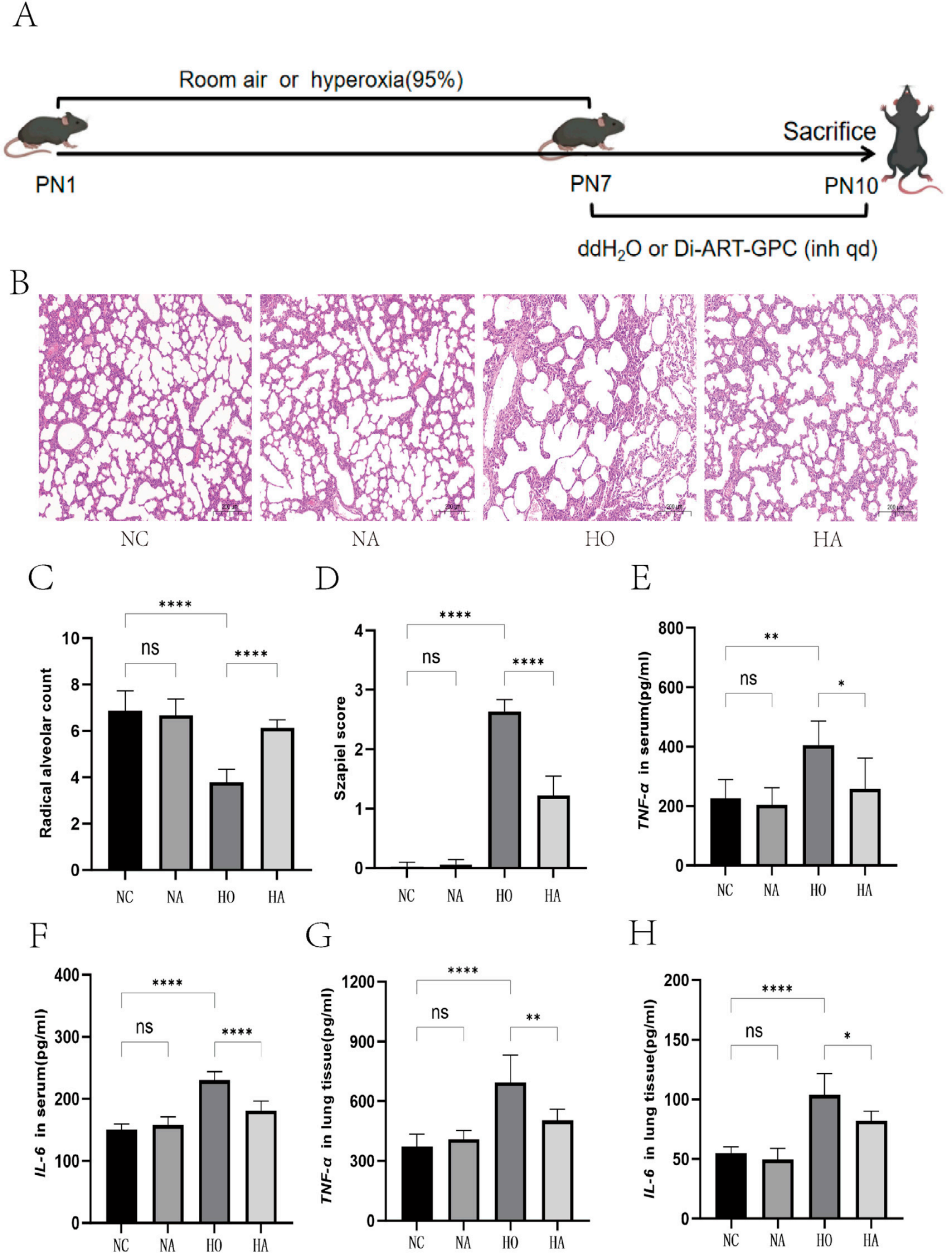
Di-ART-GPC provided protection against hyperoxia-induced lung injury in neonatal mice. **(A)** Schematic representation of the experimental procedure for the BPD model. **(B)** Hematoxylin and eosin staining of lung tissue across different groups (×100 magnification, scale bar: 200 μm). **(C)** Radical alveolar count, *n* = 6. **(D)** Alveolar injury score, *n* = 6. **(E,F)** ELISA analysis for IL-6 and TNF-α levels in serum for the indicated groups. **(G,H)** ELISA analysis for IL-6 and TNF-α levels in lung tissue for the indicated groups, *n* = 6. Data are expressed as mean ± SEM. *p < 0.05, **p < 0.01, ***p < 0.001, ****p < 0.0001 compared to the indicated group.

Neonatal C57BL/6J mice were arbitrarily allocated into four experimental cohorts: air cohort (NC), air+Di-ART-GPC cohort (NA), hyperoxia cohort (HO), and hyperoxia+Di-ART-GPC cohort (HA), with 12 mice in each cohort. The NC and NA cohorts were maintained in a normoxic environment (21% O_2_) for 7 days, while the HO and HA cohorts were continuously exposed to a hyperoxic environment (95% O_2_) for 7 days. An oxygen concentration analyzer assessed the oxygen levels within the exposure chamber, and sodium lime was employed to absorb CO_2_ produced by the mice. During this period, nursing mice alternated between high-oxygen and normal oxygen conditions to mitigate damage due to prolonged high-oxygen exposure. After 7 days, all mice were transferred to a normoxic environment and received nebulization treatment. Specifically, the mice were positioned in a transparent plastic box (30 cm × 15 cm × 15 cm) with a sidewall notch connected to the nebulizer. Each mouse received aerosolized 4 mL of ddH_2_O or 0.5 mg of Di-ART-GPC (dissolved in 4 mL ddH_2_O) for 20 min daily over three consecutive days, using a gas compression nebulizer (NE-C900, OMRON, Japan). All neonatal mice were euthanized at 11 days of age, and serum and lung tissue samples were procured for subsequent examination (Figure 1A). The experiments adhered strictly to animal ethics guidelines.

### 2.3 Histopathological evaluation

The superior lobe of the left lung underwent fixation in 4% paraformaldehyde overnight, then was embedded in paraffin and sectioned into 4 µm sequential slices. These slices were subsequently processed with HE and Masson staining at ambient temperature. Six arbitrary fields per slice were chosen for microscopic analysis (×40 magnification). Radial alveolar counts (RAC) were calculated by averaging the values, and the severity of alveolar inflammation and fibrosis was evaluated using the Szapiel et al. (1979) and Ashcroft et al. (1988) scoring systems, respectively.

### 2.4 ELISA

Blood specimens underwent centrifugation at 6,000 rpm for 15 min, and the obtained supernatant was gathered for subsequent examination. Pulmonary tissue specimens were homogenized in PBS and subjected to centrifugation at 2,000 rpm for 20 min to acquire the supernatant for assessment. TNF-α and IL-6 concentrations in serum and lung tissues were determined utilizing ELISA kits as the supplier’s protocols. Moreover, the respective ELISA kits evaluated TGF-β1, IL-6, and TNF-α levels in the cell supernatant post-centrifugation.

### 2.5 SOD, GSH, and MDA determination

SOD, GSH, and MDA assay kits, all sourced from Proteintech (Wuhan, China), were employed per the supplier’s protocols. Lung tissues were homogenized at a 1:10 (w/v) ratio, and supernatants were obtained through centrifugation at 10,000 × g at 4°C for 20 min to determine SOD, GSH, and MDA levels.

### 2.6 Immunofluorescence (IF)

Tissue slices were dewaxed, rehydrated, and submitted to heat-induced epitope retrieval. Goat serum was used for blocking for 30 min at 37°C. GPX4 primary antibody (Servicebio, GB114327, 1:3000) was incubated overnight at 4°C. Following rinsing, specimens were treated with anti-rabbit IgG fluorochrome-conjugated secondary antibody for 60 min at ambient temperature, followed by a 10-min DAPI (Servicebio, G1012,1:1000) staining process. The images were captured under a fluorescence microscope and examined with ImageJ Plus software.

### 2.7 Immunohistochemistry (IHC)

The lung tissue specimens underwent deparaffinization utilizing xylene for 30 min, then sequential rehydration in ethanol gradients (100%, 95%, 80%, 70%) and a final immersion in ddH2O for 5 min. Endogenous peroxidase was neutralized by exposing the sections to methanol comprising 3% H_2_O_2_. After three PBS washes, the specimens were blocked, comprising 5% BSA for 30 min. Primary antibodies α-SMA (Servicebio, GB191364,1:500) and TGF-β1 (Servicebio, GB11179,1:500) were applied and incubated overnight at 4°C. After rinsing, the sections underwent incubation with a biotinylated secondary antibody for 30 min at 37°C. DAB served as the chromogen, while hematoxylin was utilized as the counterstain. Lastly, the specimens were dehydrated, cleared in xylene, and mounted with neutral balsam. Histological images were captured utilizing a Nikon digital sight imaging system connected to a Nikon E-600 microscope (Nikon, Japan).

### 2.8 Real-time quantitative polymerase chain reaction (RT-qPCR)

Total RNA was procured from lung tissues and RAW264.7 cells utilizing TRIzol reagent (Invitrogen, United States), and cDNA synthesis was performed using a reverse transcription kit (Proteinbio, Nanjing, China). The qPCR reaction mixture comprised 12.5 µL 2 × SYBR qPCR Mix, 1 µL cDNA, 1 µL forward primer (10 μmol/L), and 1 µL reverse primer (10 μmol/L), as per the polymerase chain reaction kit’s instructions (Proteinbio, Nanjing, China). The volume was brought to 25 µL with ddH2O. The qPCR protocol involved an initial denaturation at 95°C for 2 min, succeeded by 40 cycles at 95°C for 15 s and 60°C for 30 s. Reactions were executed using a LightCycler 480 real-time PCR system (Roche, United States). The primer sequences utilized were as follows:

TGF-β1: Forward: CCT​GGA​CAC​ACA​GTA​CAG​CA; Reverse: CCA​CGT​AGT​AGA​CGA​TGG​GC

α-SMA: Forward: 5′-CAT​CCG​ACC​TTG​CTA​ACG​GA-3’; Reverse: 5′-CCA​CAT​ACA​TGG​CAG​GGA​CA-3′

GPX4: Forward: 5′-GGC​AGG​AGC​CAG​GAA​GTA​AT-3’; Reverse: 5′-ACC​AGC​CGT​TCT​TAT​CAA​T-3′

GAPDH: Forward: 5′-CCG​CAT​CTT​CTT​GTG​CAG​TG-3’; Reverse: 5′-TAC​GGC​CAA​ATC​CGT​TCA​CA-3’.

### 2.9 Cell culture and treatment

RAW264.7 cells, procured from the American Type Culture Collection (Rockville, MD, United States), were kept in DMEM supplemented with 10% fetal bovine serum and 1% antibiotics in a moisture-controlled incubator with 5% CO_2_ at 37°C. Cells were placed in six-well plates at 1 × 10^6^ cells/mL and incubated in a serum-free medium for 12 h. The experiment involved four cohorts: NC, NA, HO, and HA. The NC and NA cohorts were cultured in a constant-temperature incubator maintained at 37°C with 5% CO_2_, while the HO and HA cohorts were maintained in a hyperoxic incubator with 95% O_2_ for 24 h. In the HA cohort, 1.25 µM of Di-ART-GPC was added. Cells and culture supernatants were collected for subsequent analysis of relevant markers (Figure 4A).

### 2.10 CCK-8 assay

RAW264.7 cells (5 × 10^3^/well) were placed in 96-well plates and left overnight in 100 µL of medium to assess the cytotoxicity of Di-ART-GPC. Following the initial incubation, the medium was exposed to various concentrations of Di-ART-GPC (0.625, 1.25, 2.5, 5, 10, and 20 µM). After 12 and 24 h of treatment, 10 µL of CCK-8 reagent was introduced to each well and kept in darkness for 1 h. Absorbance (OD) was ascertained at 450 nm utilizing a microplate reader. Each experiment was conducted in triplicate.

### 2.11 Protein extraction and western blot (WB)

For protein examination, cells underwent lysis in RIPA buffer with 1% PMSF added, and total cellular proteins were isolated and measured utilizing a BCA assay kit (Beyotime, Shanghai, China). Proteins were divided by SDS-PAGE and moved to PVDF membranes (Meck Millipore, Burlington, United States). Following blockage with 5% milk for 1 h at ambient temperature, the membranes were exposed to primary antibodies at 4°C. The primary antibodies employed included GPX4 (Servicebio, GB15001, 1:2000) and ACTIN (Servicebio, GB113745, 1:2000). Membranes were subsequently exposed to horseradish peroxidase-linked secondary antibodies for 1 h at room temperature. Protein bands were detected using enhanced chemiluminescence (ECL) and evaluated with ImageJ software. Three independent samples were tested for each cohort.

### 2.12 Statistical methods

All statistical evaluations were executed utilizing GraphPad Prism 10 (San Diego, CA, United States). Results were denoted as mean ± SEM. One-way analysis of variance (ANOVA) was employed to examine the data, succeeded by Tukey’s *post hoc* test for inter-group comparisons. A p-value below 0.05 was deemed statistically significant.

## 3 Results

### 3.1 Di-ART-GPC ameliorates lung inflammation in hyperoxia-exposed neonatal mice

HE staining exhibited typical alveolar morphology, well-defined alveolar walls, and no significant inflammatory cell infiltration in the NC and NA cohorts, with no observable differences between them. In contrast, the HO cohort, following hyperoxia exposure, displayed enlarged alveoli with a reduced number, substantial inflammatory cell infiltration, interstitial edema, and thickened alveolar walls. These pathological changes were ameliorated after DI-ART-GPC intervention (Figure 1B). The analysis of RAC values showed marked differences across the four cohorts (F = 28.64, p < 0.001). In contrast to the NC cohort, the HO cohort displayed markedly reduced RAC values (p < 0.05).

Nevertheless, the RAC measurements in the HA cohort were substantially elevated compared to the HO cohort (p < 0.05) and exhibited no significant difference from the NC cohort (Figure 1C). Regarding inflammation indices, substantial differences were observed among the four cohorts (F = 232, p < 0.001), with HO and HA cohorts displaying markedly elevated scores relative to the NC cohort (both p < 0.05). However, the inflammation index was considerably lower in the HA cohort compared to the HO cohort (p < 0.05) (Figure 1D).

### 3.2 Di-ART-GPC reduces inflammatory cytokine levels in lung tissue and serum of hyperoxia-exposed neonatal mice

The ELISA findings revealed comparable concentrations of IL-6 and TNF-α in the NC and NA cohorts for both serum and lung tissue homogenates. However, IL-6 and TNF-α levels were markedly elevated in the HO and HA cohorts relative to the NA cohort (p < 0.05). These levels were markedly reduced in the HA cohort relative to the HO cohort (p < 0.05) (Figures 1E–H).

### 3.3 Di-ART-GPC reduces lung fibrosis levels in hyperoxia-exposed neonatal mice

**FIGURE 2:**
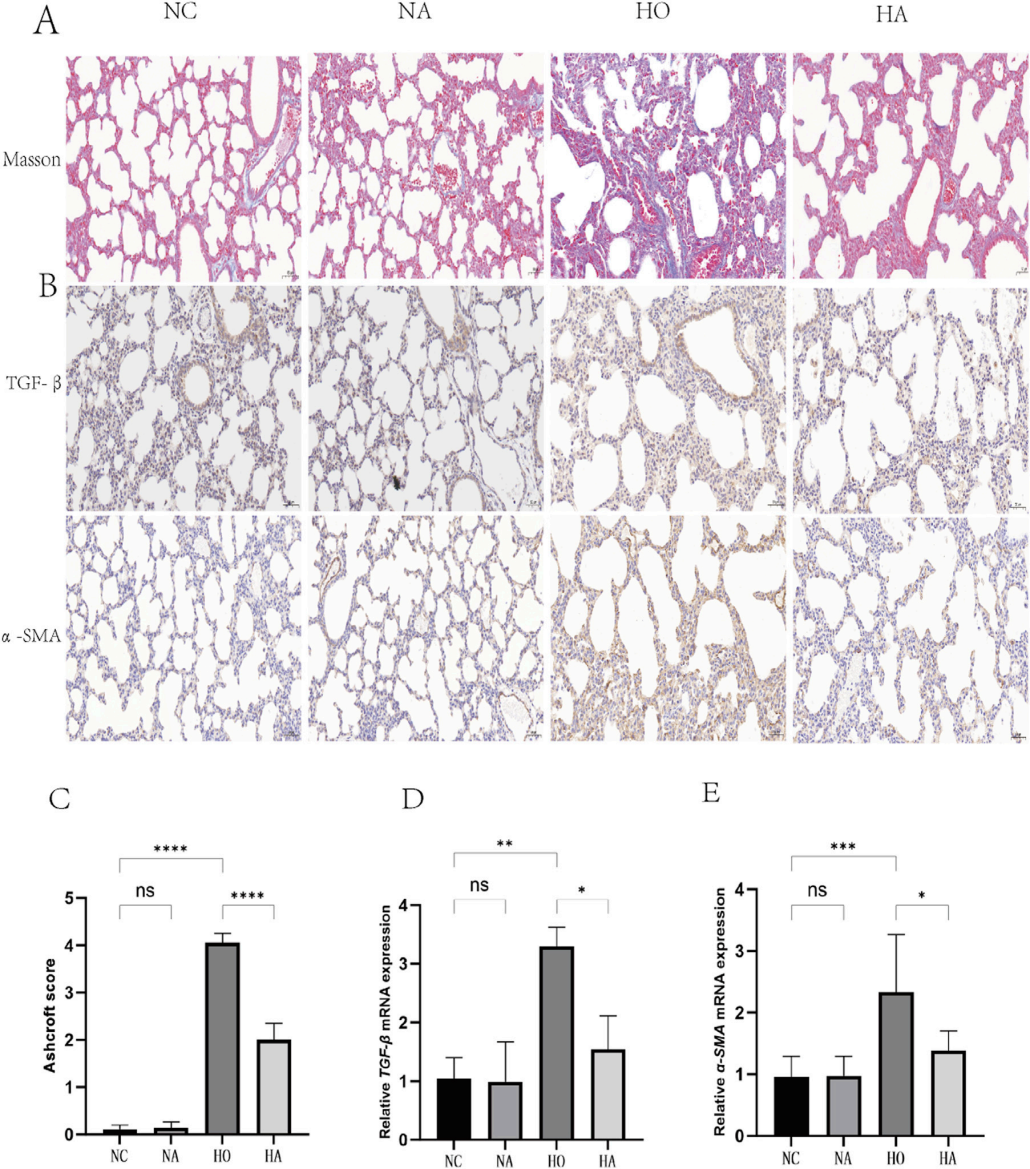
Di-ART-GPC attenuated hyperoxia-induced fibrosis in neonatal mice. **(A)** Masson staining of lung tissue across groups (×200 magnification, scale bar: 100 µm). **(B)** Immunohistochemical (IHC) staining of TGF-β1 and α-SMA in lung sections (×400 magnification, scale bar:50 µm). **(C)** Fibrosis scores of lung tissue (*n* = 6). **(D,E)** Relative mRNA expression levels of TGF-β1 and α-SMA (*n* = 3). Data are presented as mean ± SEM. *p < 0.05, **p < 0.01, ***p < 0.001, ****p < 0.0001, compared to the indicated group.

As illustrated in Figure 2A, masson staining indicated that the alveolar structure in the NC and NA cohorts was intact, with clear alveolar walls and a minimal presence of blue collagen fibers around the bronchioles and alveolar septa. The HO cohort, showed a marked increase in collagen fiber content and alveolar septal thickening relative to the NC cohort. Conversely, the HA cohort exhibited a notable reduction in collagen fiber content and a diminishment in lung fibrosis relative to the HO cohort. The differences in fibrosis scores across the four cohorts were significant (F = 453, p < 0.001), with both the HO and HA cohorts displaying markedly higher fibrosis scores than the NC cohort (both p < 0.05). However, the fibrosis score in the HA cohort was markedly lower than that of the HO cohort (p < 0.05) (Figure 2C).

### 3.4 Di-ART-GPC downregulated TGF-β1 and α-SMA expression in lungs of hyperoxia-exposed neonatal mice

The abundance of TGF-β1 and α-SMA were assessed utilizing immunohistochemistry and RT-qPCR. No significant differences were noted between the NC and NA cohorts. Nevertheless, compared to the NC cohort, the HO cohort exhibited elevated levels of the pro-fibrotic factor TGF-β1 and the fibrotic marker α-SMA in the lungs (p < 0.05). Conversely, the HA cohort exhibited markedly diminished levels of TGF-β1 and α-SMA compared to the HO cohort (p < 0.05) (Figures 2B,D,E).

### 3.5 Di-ART-GPC suppressed ferroptosis in hyperoxia-exposed neonatal mice

**FIGURE 3:**
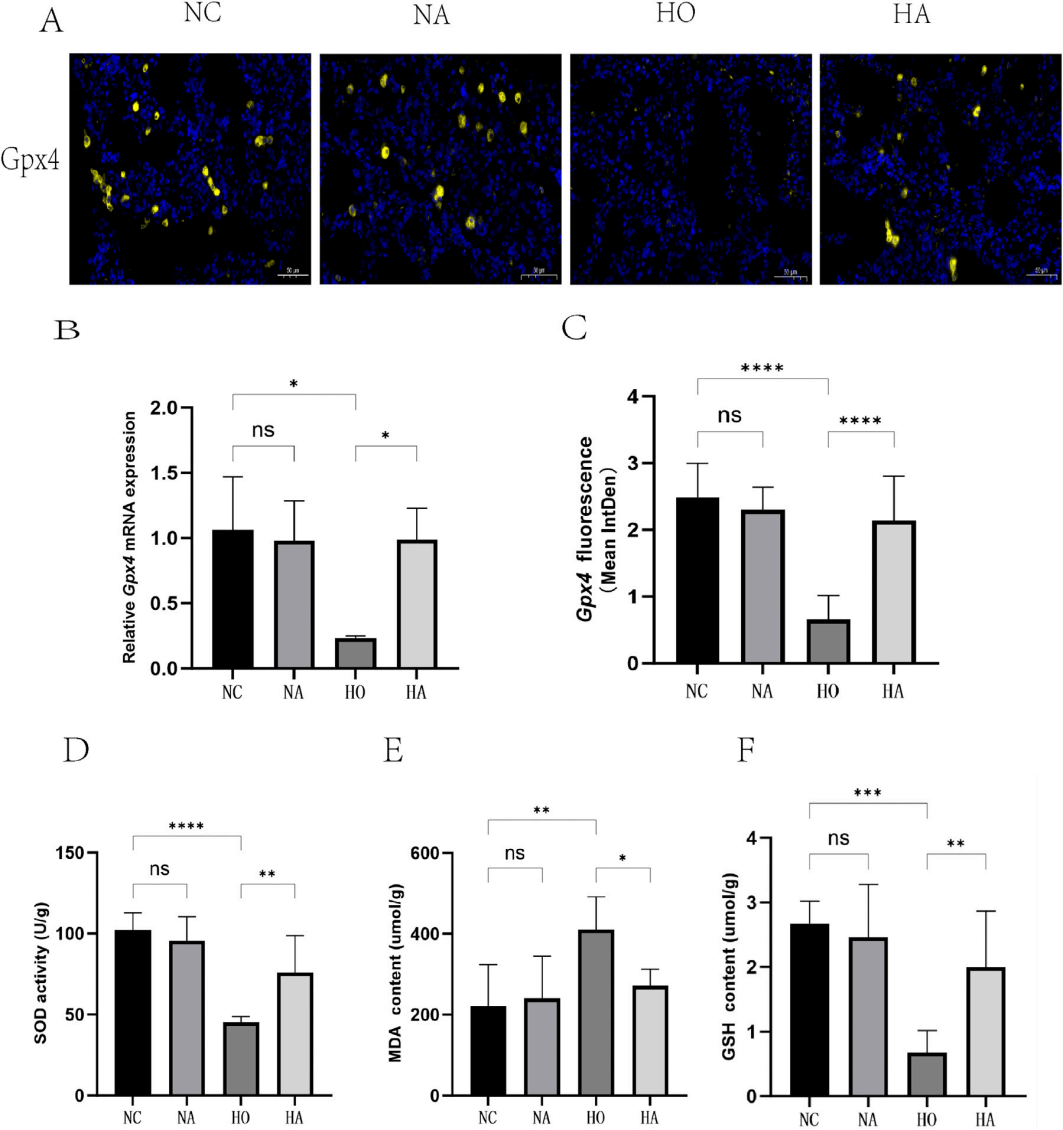
Di-ART-GPC significantly inhibited ferroptosis in neonatal mice following hyperoxia-induced lung injury. **(A)** Representative immunofluorescence images of GPX4 in lung tissues, with yellow indicating GPX4 staining and blue indicating nuclear staining (×400 magnification, scale bar: 50 µm). **(B)** Relative mRNA expression of GPX4 (*n* = 3). **(C)** Mean fluorescence intensity of GPX4 across different groups. **(D–F)** Activities of SOD, MDA, and GSH in lung tissues. Data are expressed as mean ± SEM. *p < 0.05, **p < 0.01, ***p < 0.001, ****p < 0.0001, compared to the indicated group.

The expression level of GPX4, a critical inhibitor of ferroptosis in lung tissue, was evaluated using immunofluorescence and RT-qPCR. No significant difference in GPX4 expression was observed between the NC and NA cohorts. However, the immunofluorescence quantification and mRNA expression levels of GPX4 in the HO cohort were considerably diminished compared to the NC cohort (p < 0.05). Conversely, the HA cohort exhibited a substantial elevation in GPX4 levels relative to the HO cohort (p < 0.05) (Figures 3A–C).

Furthermore, the levels of the lipid peroxidation product MDA, the antioxidant enzyme SOD, and the antioxidant GSH were assessed. The results indicated that MDA levels were markedly elevated (p < 0.05), while SOD and GSH levels were markedly reduced (p < 0.05) in the lung tissues of the HO cohort. In comparison, the HA cohort exhibited decreased MDA levels and markedly elevated SOD and GSH levels (p < 0.05) relative to the HO cohort (Figures 3D–F).

### 3.6 Cytotoxic effect of Di-ART-GPC on RAW264.7 cells

**FIGURE 4:**
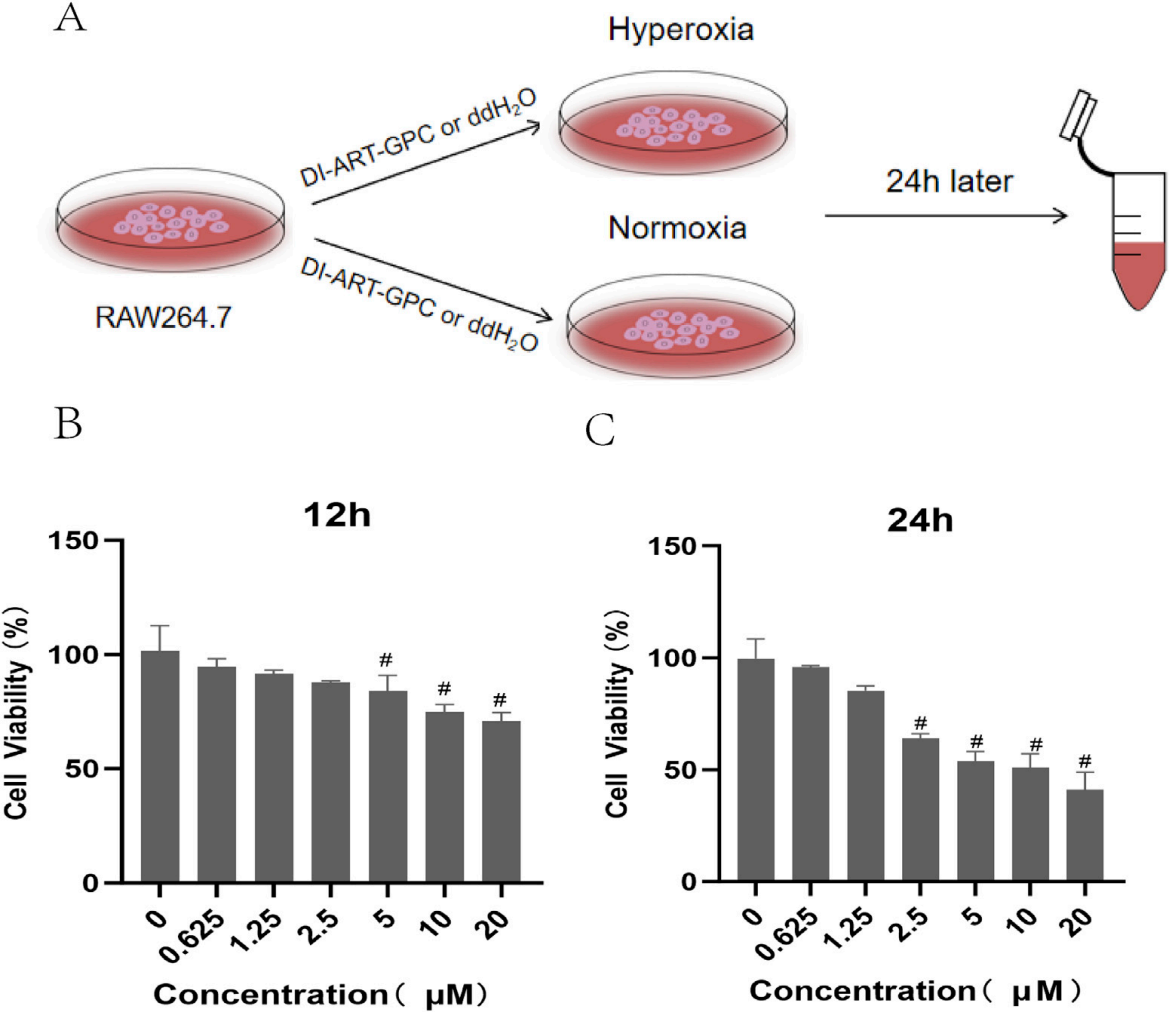
Effects of Di-ART-GPC treatment on the viability of RAW264.7 cells. **(A)** Experimental procedure for RAW264.7 cells. **(B,C)** CCK-8 assay results for RAW264.7 cells following 12/24 h Di-ART-GPC treatment at varying doses. Data are presented as mean ± SEM. #p < 0.05, compared to the control group.

To evaluate the cytotoxicity of Di-ART-GPC on RAW264.7 cells, the cells were exposed to escalating doses of Di-ART-GPC (0.625, 1.25, 2.5, 5, 10, and 20 µM) for 12 and 24 h. Cell viability was determined utilizing the CCK-8 assay. The outcomes demonstrated no significant cytotoxic effects at concentrations from 0 to 1.25 µM (Figures 4B,C). Consequently, 1.25 µM was selected for subsequent experiments.

### 3.7 Di-ART-GPC successfully reduced the levels of IL-6, TNF-α and TGF-β1 in hyperoxia-exposed RAW264.7 cells

**FIGURE 5:**
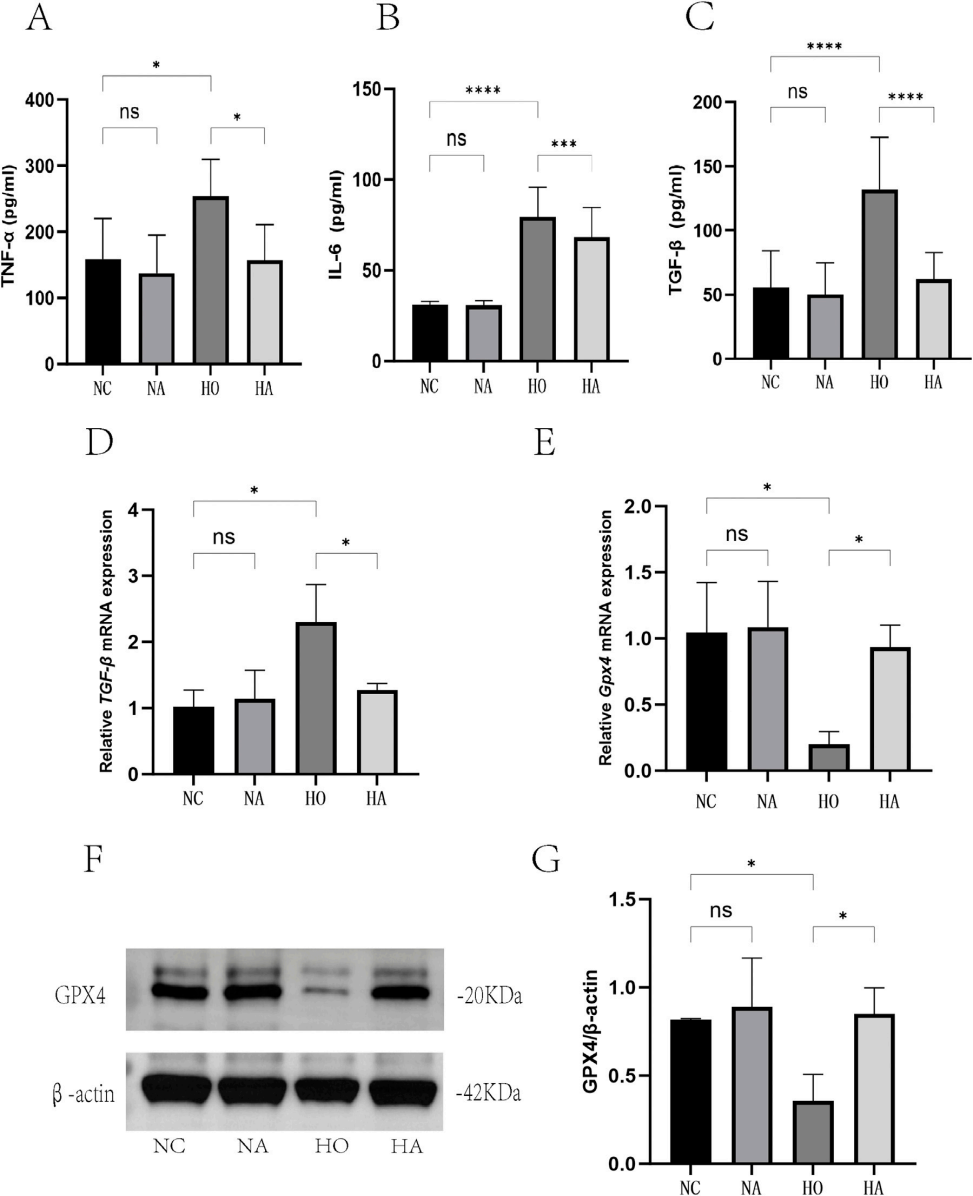
Di-ART-GPC suppressed the expression of TNF-α, IL-6, TGF-β1, and GPX4 in RAW264.7 cells. **(A–C)** Levels of TNF-α, IL-6, and TGF-β1 in the supernatant of RAW264.7 cells (*n* = 6). **(D)** Relative mRNA expression of TGF-β1 (*n* = 3). **(E)** Relative mRNA expression of GPX4 (*n* = 3). **(F)** Protein levels of GPX4 in RAW264.7 cells. **(G)** Quantitative analysis of GPX4 protein expression (*n* = 3). Data are expressed as mean ± SEM. *p < 0.05, **p < 0.01, ***p < 0.001, ****p < 0.0001, compared to the indicated group.

The concentrations of cytokines in the RAW264.7 cell supernatant were assessed by quantifying the expression of TNF-α, IL-6, and TGF-β1. No notable variations were detected between the NC and NA cohorts. However, compared to the NC cohort, the HO cohort exhibited markedly increased levels of the inflammatory indicators TNF-α and IL-6, as well as the fibrosis-promoting factor TGF-β1 (p < 0.05). Conversely, the HA cohort demonstrated markedly lower levels of these markers when compared to the HO cohort (p < 0.05) (Figures 5A–D).

### 3.8 Di-ART-GPC inhibits ferroptosis in hyperoxia-exposed RAW264.7 cells

The expression of the ferroptosis inhibitor GPX4 in RAW264.7 cells was examined utilizing RT-qPCR and WB. No significant difference was noted between the NC and NA cohorts. However, GPX4 mRNA and protein levels were markedly lower in the HO cohort relative to the NC cohort (p < 0.05), while they were markedly higher in the HA cohort relative to the HO cohort (p < 0.05) (Figures 5E–G).

## 4 Discussion

BPD is a severe chronic lung disease primarily impacting preterm infants, for which effective treatments remain lacking (Poets and Lorenz, 2018). Artemisinin, a plant-derived compound known for its broad biological activities, has demonstrated therapeutic potential across various diseases (Wu et al., 2020). Our investigation examined the impact of Di-ART-GPC in a hyperoxia-induced BPD model. Our findings revealed that Di-ART-GPC, administered via aerosol inhalation, markedly reduced inflammatory factor levels, alleviated inflammatory injury, and mitigated fibrosis in the lungs of BPD mice. Furthermore, it enhanced the activity of the antioxidant enzyme SOD, increased GSH content, and upregulated GPX4 protein expression. *In vitro*, Di-ART-GPC decreased inflammatory factor levels in hyperoxia-exposed macrophages while also elevating GPX4 expression, suggesting that Di-ART-GPC may protect against BPD lung injury by regulating macrophage ferroptosis.

The role of inflammatory processes in the development of hyperoxia-induced BPD is well-established. Hyperoxia activates inflammatory pathways, leading to the infiltration of immune cells, encompassing macrophages and neutrophils, which subsequently cause lung injury and disrupt normal lung development (Buczynski et al., 2013; Kalikkot Thekkeveedu et al., 2017). As the most abundant immune cells in lung tissues, alveolar macrophages mediated lung injury by synthesizing and releasing pro-inflammatory mediators (Zhou et al., 2020). After hyperoxia exposure, the number of alveolar macrophages increases markedly, leading to alveolar damage via the secretion of pro-inflammatory mediators, encompassing TNF-α and IL-6, which hinders the development of immature lungs (Ma et al., 2023; Kalymbetova et al., 2018). Prior investigations have demonstrated that artemisinin can mitigate acute lung injury by modulating various inflammatory signaling pathways (Efferth and Oesch, 2021; Zhang et al., 2022). For example, You et al. found that artemisinin reduces TNF-α, IL-6, and TGF-β1 levels, inhibiting inflammatory responses and fibrosis by regulating the TGF-β/JAK2/STAT3 signaling pathway (You et al., 2022). Weng et al. reported that artemisinin alleviates hyperoxia-induced BPD injury by inhibiting NF-κB pathway activation and inflammatory factor expression (Weng et al., 2024). Consistent with these findings, our study demonstrated that hyperoxia exposure in BPD model mice resulted in markedly elevated TNF-α and IL-6 levels, alongside pronounced inflammatory cell infiltration, edema, and alveolar septal thickening. After Di-ART-GPC intervention, these mice exhibited reduced levels of inflammatory cytokines, lower alveolar injury scores, and improved pathological outcomes. *In vitro*, Di-ART-GPC also markedly reduced IL-6 and TNF-α secretion in hyperoxia-cultured macrophages, suggesting that it mitigates hyperoxia-induced lung injury in BPD mice by inhibiting macrophage-mediated inflammatory responses. In contrast to existing studies, our *in vitro* macrophage assays specifically demonstrate the crucial involvement of macrophages in BPD progression.

The late stage of BPD is primarily marked by abnormal lung tissue repair, which leads to interstitial fibrosis (Hwang and Rehan, 2018). TGF-β1, a crucial mediator of lung developmental abnormalities, plays a pivotal role in inducing fibroblast migration, transforming normal fibroblasts into myofibroblasts, promoting extracellular matrix (ECM) deposition, and enhancing collagen expression while inhibiting its degradation (Molagoda et al., 2023). This process leads to an increase in the fibrosis marker protein α-SMA (Yanagihara et al., 2022). Recent research has emphasized the importance of macrophages in promoting fibroblast proliferation, differentiation, and collagen synthesis, via TGF-β1 alone or in conjunction with inflammatory factors (Su et al., 2020; Lodyga and Hinz, 2020; Bolourani et al., 2021). Artemisinin has repeatedly been shown to possess anti-fibrotic properties, with numerous studies indicating that it may mitigate pulmonary fibrosis by inhibiting fibroblast proliferation (Dolivo et al., 2021). For instance, artemisinin has been found to inhibit myofibroblast growth and reduce bleomycin-induced pulmonary fibrosis by downregulating pro-fibrotic proteins such as TGF-β1, Smad3, HSP47, α-SMA, and collagen type I (Wang et al., 2015; Yang et al., 2015). Consistent with these observations, our research demonstrated that Di-ART-GPC improved lung pathology in BPD mice, particularly by reducing fibrosis levels and decreasing TGF-β1 and α-SMA levels. Moreover, *in vitro* experiments revealed that Di-ART-GPC markedly reduced TGF-β1 secretion in hyperoxia-exposed macrophages, indicating its protective effect against hyperoxia-induced pulmonary fibrosis.

Ferroptosis, an iron-reliant form of regulated cellular demise triggered by lipid oxidation and reactive oxygen species buildup (Li et al., 2020), has been linked to the onset of various disorders (Xie et al., 2016). Research has shown that this process is intimately connected to the progression of BPD (Chen et al., 2023). Premature infants, who are often affected by BPD, are especially susceptible to oxidative damage owing to elevated high oxygen exposure and immature antioxidant systems, leading to ferroptosis (Lan et al., 2023). Consistent with the literature, our study found that neonatal mice exposed to high-oxygen environments exhibited signs of ferroptosis and impaired lung development (Yang et al., 2023; Chou and Chen, 2022b). GPX4, a selenoprotein that mitigates lipid oxidative damage, is a key upstream regulator of ferroptosis (Liu Y. et al., 2023). GPX4 utilizes the antioxidant GSH as a substrate to reduce hydroperoxides, including phospholipid and cholesterol hydroperoxides, thereby lowering lipid ROS production and preventing ferroptosis (Seibt et al., 2019). Cell ferroptosis is typically accompanied by a decline in the antioxidant systems GSH and GPX4, along with the accumulation of lipid peroxides (Yan et al., 2020; Liu J. et al., 2023). A recent study demonstrated that artemisinin alleviates radiation-induced lung injury by modulating the Nrf2/HO-1 pathway, thereby reducing cellular ferroptosis (Ning et al., 2024). In our study, BPD model mice exposed to hyperoxia showed diminished levels of the antioxidant enzymes SOD, GSH, and GPX4 alongside elevated MDA content. Treatment with Di-ART-GPC markedly increased SOD, GSH, and GPX4 levels while decreasing MDA content, suggesting that Di-ART-GPC inhibits ferroptosis in lung tissue cells. Ferroptosis is also present in macrophages and contributes to lung injury caused by various factors. Inhibiting macrophage ferroptosis has been shown to attenuate lung inflammation effectively (Cao et al., 2023; Yang et al., 2022; Xu et al., 2024). In our study, Di-ART-GPC increased GPX4 mRNA and protein expression in hyperoxia-exposed macrophages *in vitro*, suggesting that it may inhibit macrophage ferroptosis and contribute to the protection against lung injury.

Currently, the primary routes of administration for artemisinin in clinical applications include oral, rectal, intravenous, and intramuscular delivery (Ruwizhi et al., 2022). In preclinical *in vivo* studies, the drug is commonly administered via gavage, intraperitoneal, and intravenous routes. However, for respiratory diseases, aerosol inhalation has emerged as an efficient method for delivering the drug directly to the lungs while minimizing systemic exposure. This method helps to avoid the systemic side effects often associated with oral or other administration routes. Moreover, aerosol inhalation is non-invasive, posing minimal infection risk, which is particularly advantageous for neonates and small infant patients in clinical settings (Elkasabgy et al., 2020). In our study, we utilized artemisinin-loaded liposomes, which facilitated cellular drug entry, enhancing the anti-inflammatory effects and improving both the concentration and bioavailability of the drug in the lungs, ultimately leading to superior therapeutic outcomes (Adel et al., 2021). Recent studies on asthma and acute lung injury have demonstrated that artemisinin can exert significant therapeutic effects when inhaled aerosol (Hu et al., 2016; Wang et al., 2019).

In conclusion, our findings indicate that Di-ART-GPC exhibits a safeguarding influence on hyperoxia-induced lung injury in BPD mice, likely due to its ability to reduce inflammation and fibrosis, and inhibit macrophage ferroptosis in the lungs. Using aerosol inhalation as a delivery method for Di-ART-GPC offers a promising new approach for its clinical application. However, this study has some limitations. Specifically, in the *in vivo* experiments, no dose gradient of artemisinin was explored to assess dose-dependent effects, and the role of ferroptosis in lung macrophages was not studied in isolation. Further investigation into the specific mechanisms of ferroptosis and the therapeutic potential of Di-ART-GPC inhalation in BPD treatment may pave the way for new strategies for managing BPD.

## Data Availability

The original contributions presented in the study are included in the article/Supplementary Material, further inquiries can be directed to the corresponding authors.
