# Supplementary figures and images for: Aerosol inhalation of dimeric artesunate phospholipid-conjugated liposomes ameliorates inflammation, fibrosis, and ferroptosis in neonatal mice with hyperoxia-induced lung injury
Source: Front Pharmacol. 2025 Jul 21;16:1542743. doi: 10.3389/fphar.2025.1542743 (PMC12319227; doi:10.3389/fphar.2025.1542743)

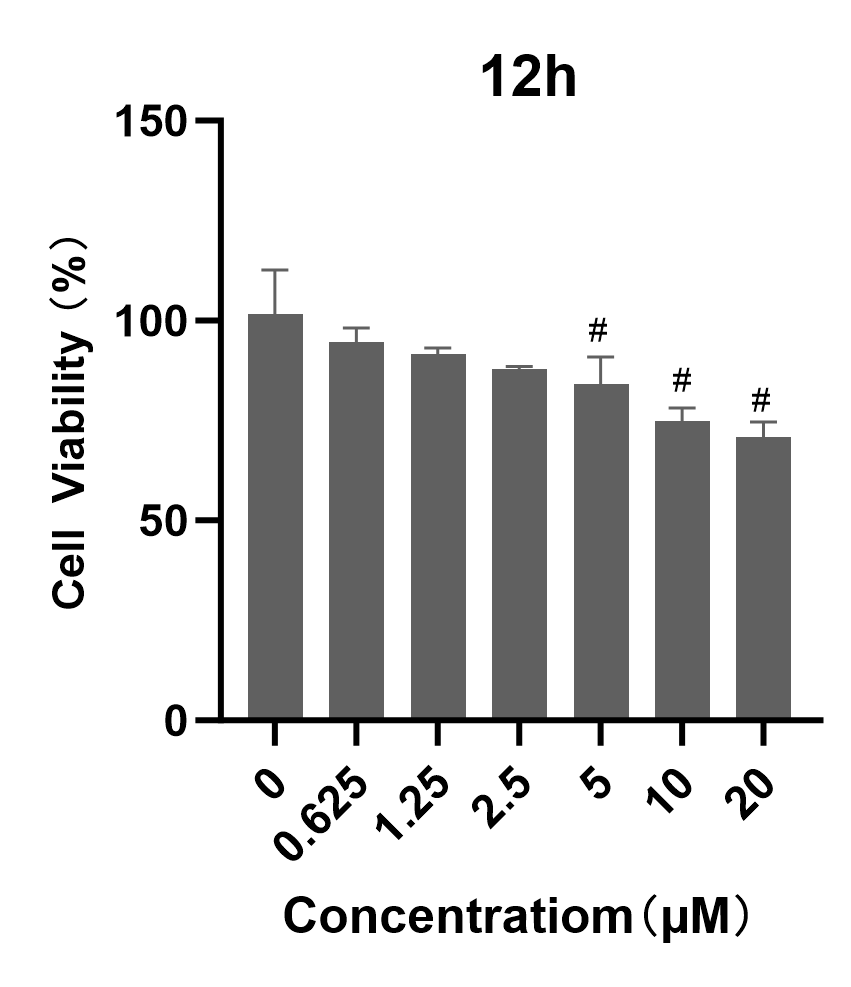

Supplement: Supplementary file 1 [file DataSheet1.zip › data and figures/CCK8/CCK8 12h.tif]

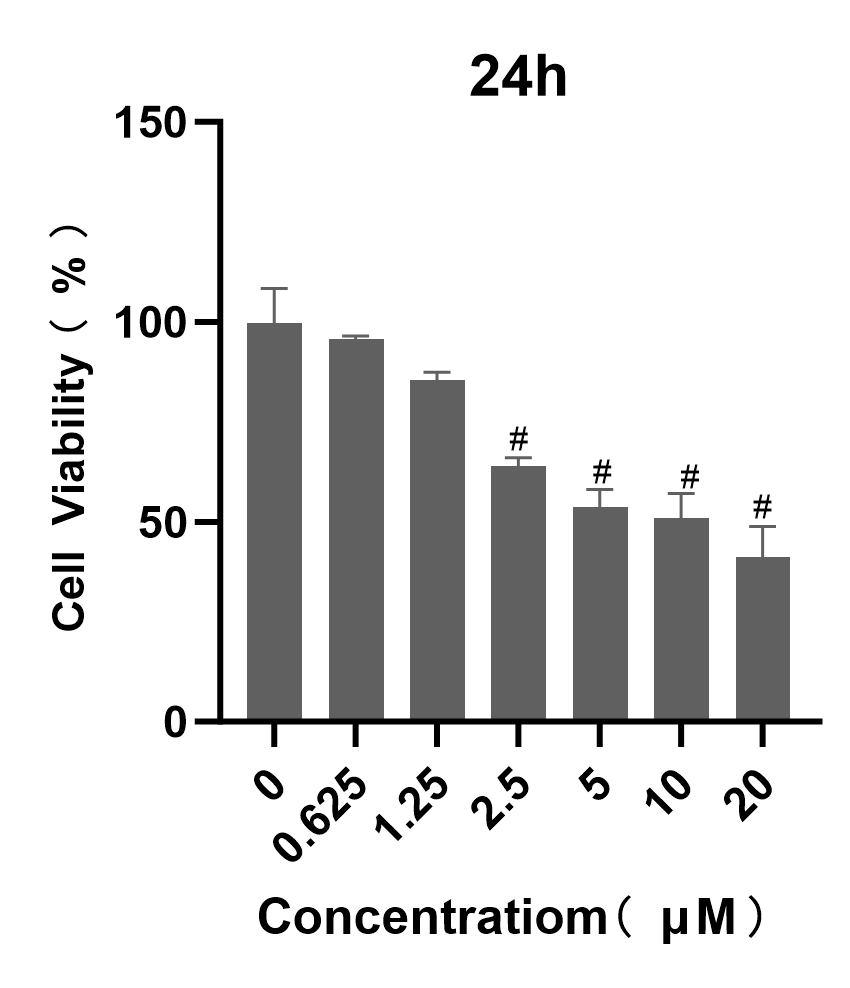

Supplement: Supplementary file 1 [file DataSheet1.zip › data and figures/CCK8/CCK824h.tif]

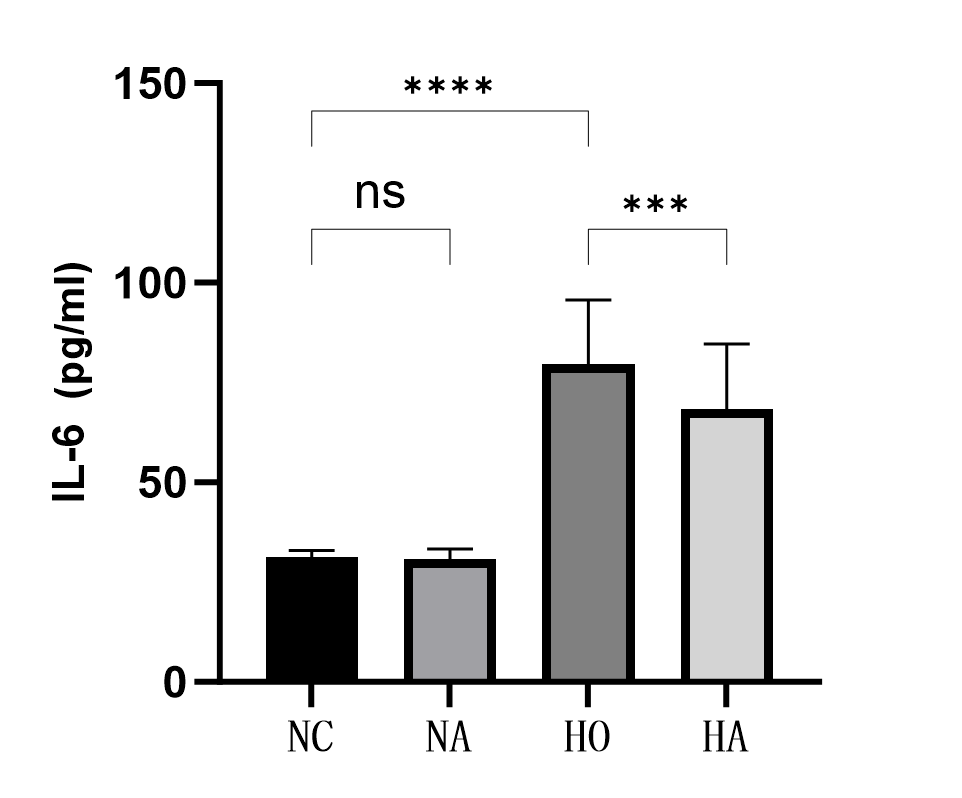

Supplement: Supplementary file 1 [file DataSheet1.zip › data and figures/ELISA/IL-6 in cell.tif]

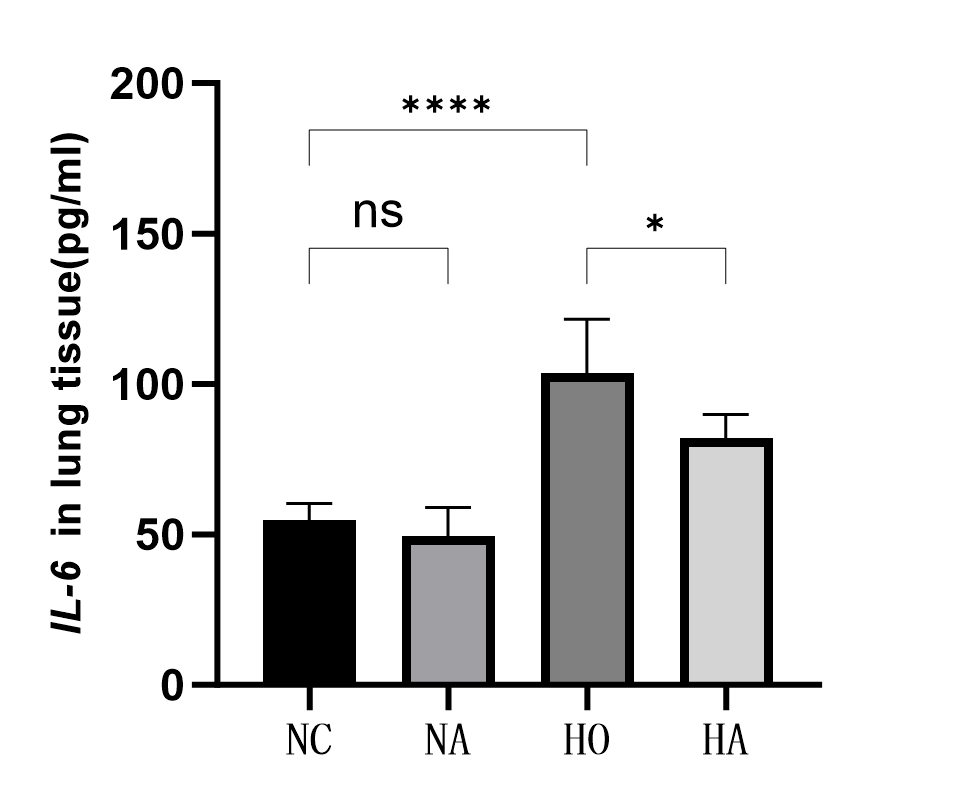

Supplement: Supplementary file 1 [file DataSheet1.zip › data and figures/ELISA/IL-6 in lung.tif]

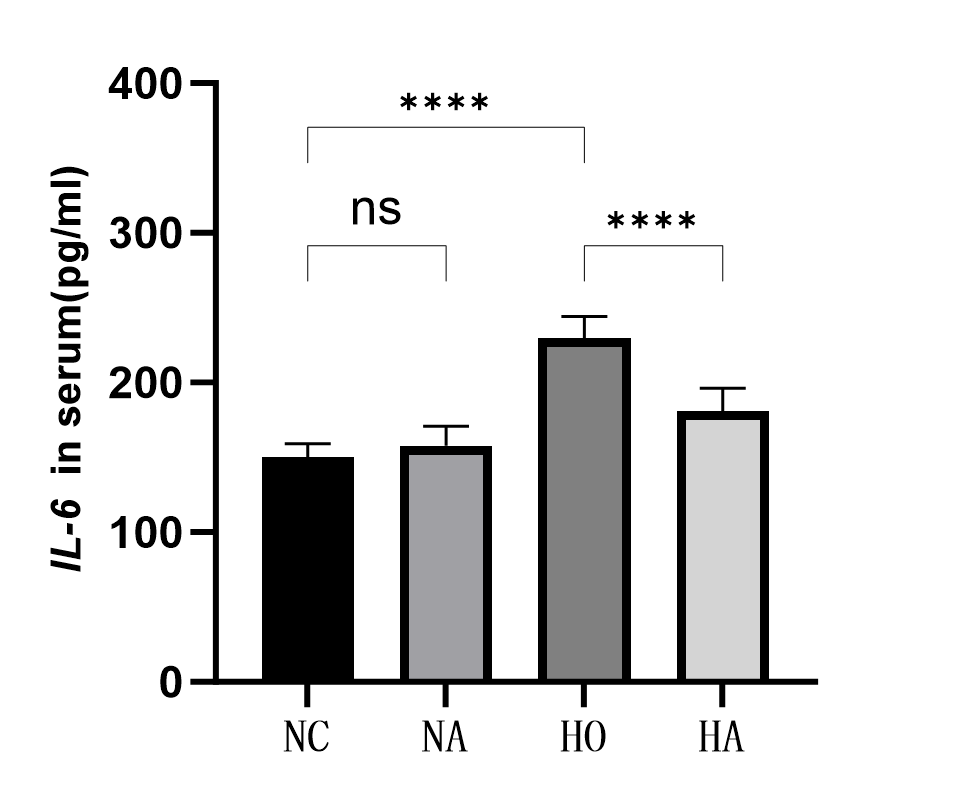

Supplement: Supplementary file 1 [file DataSheet1.zip › data and figures/ELISA/IL-6 in serum.tif]

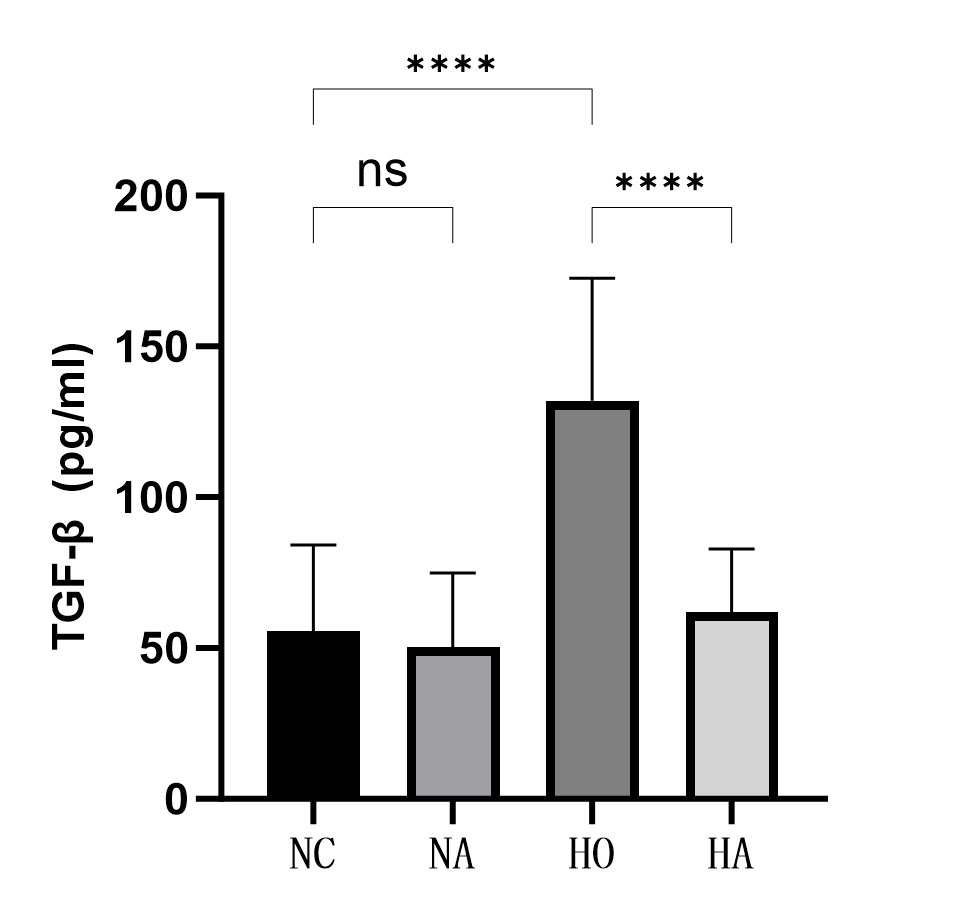

Supplement: Supplementary file 1 [file DataSheet1.zip › data and figures/ELISA/TGF-a┬ in cell.tif]

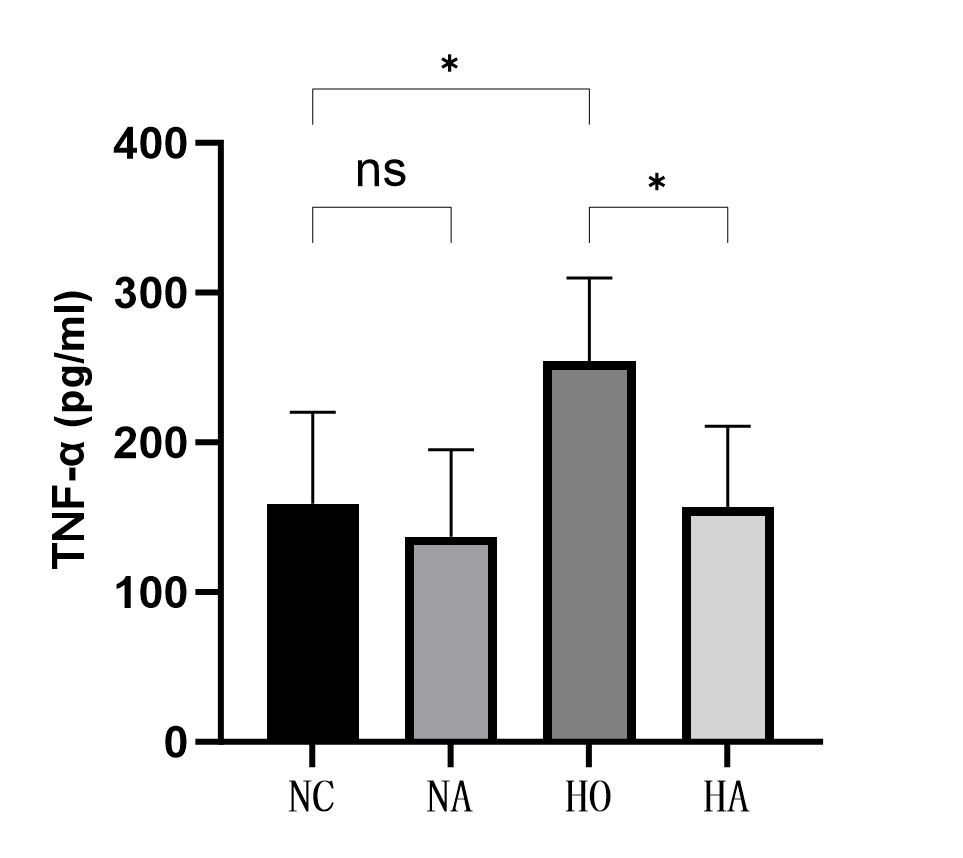

Supplement: Supplementary file 1 [file DataSheet1.zip › data and figures/ELISA/TNF-a┴ in cell.tif]

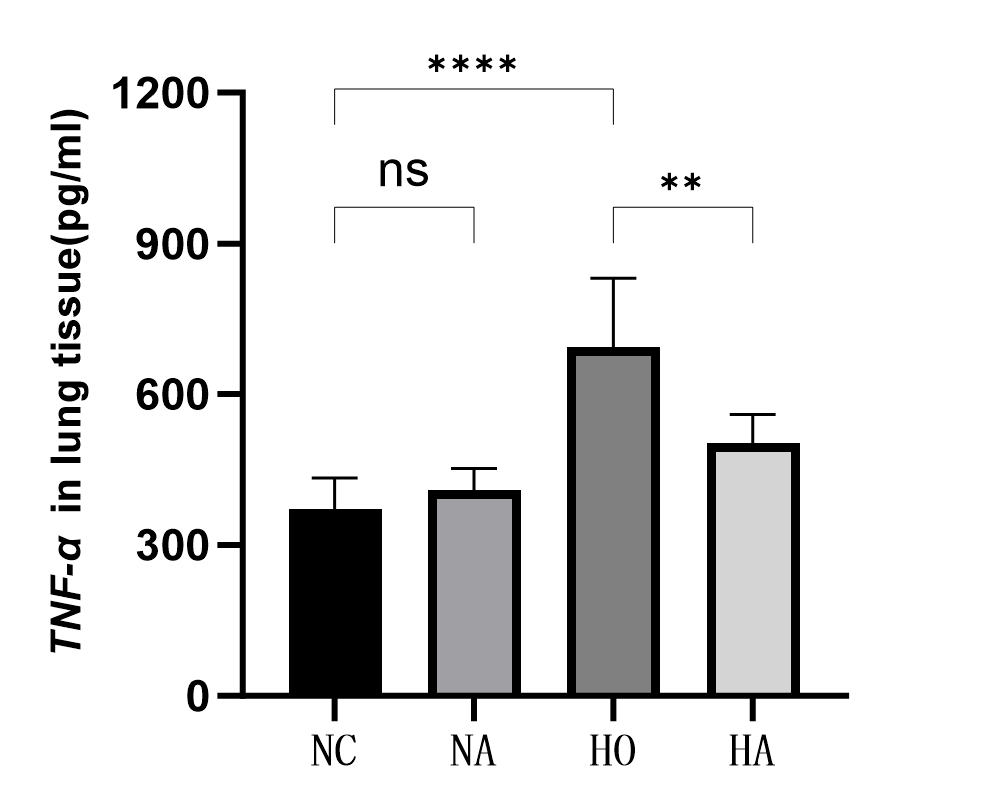

Supplement: Supplementary file 1 [file DataSheet1.zip › data and figures/ELISA/TNF-a┴ in lung.tif]

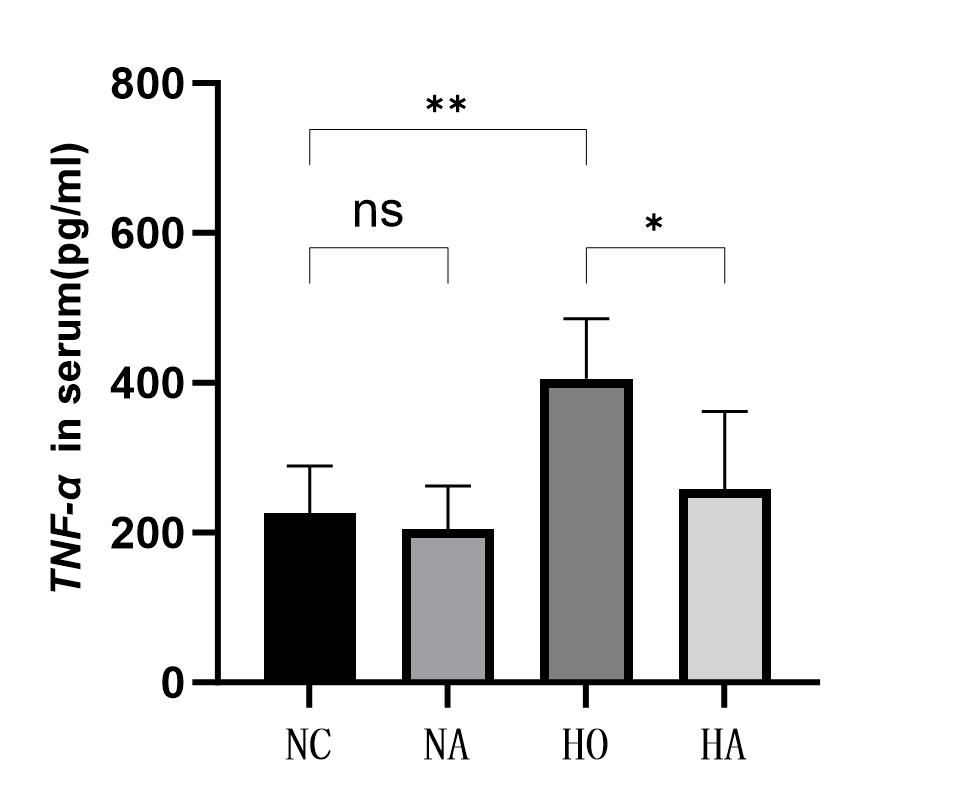

Supplement: Supplementary file 1 [file DataSheet1.zip › data and figures/ELISA/TNF-a┴ in serum.tif]

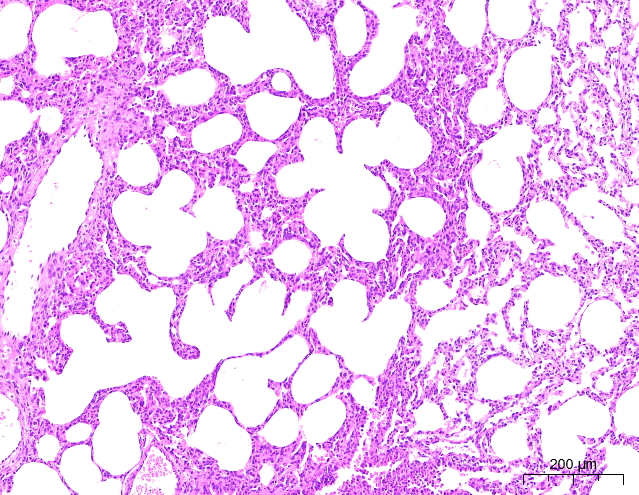

Supplement: Supplementary file 1 [file DataSheet1.zip › data and figures/HE/ho.jpg]

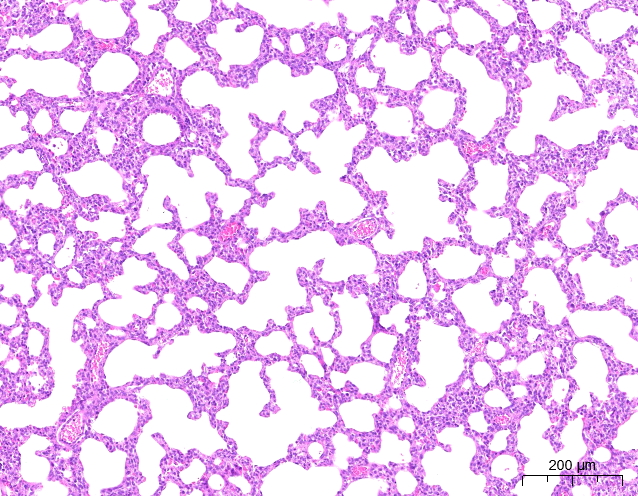

Supplement: Supplementary file 1 [file DataSheet1.zip › data and figures/HE/ho+a.jpg]

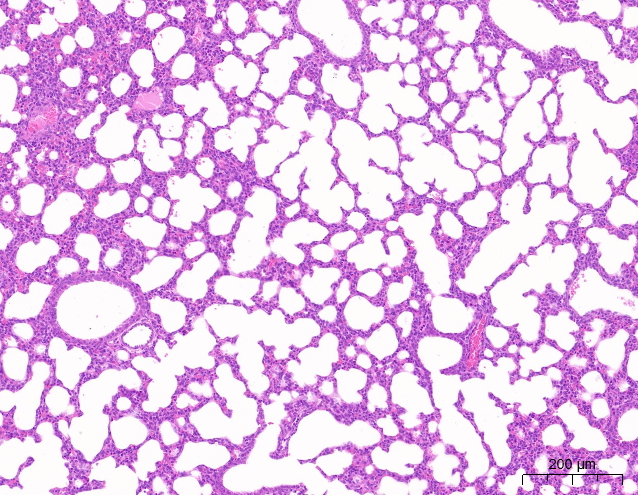

Supplement: Supplementary file 1 [file DataSheet1.zip › data and figures/HE/nc.jpg]

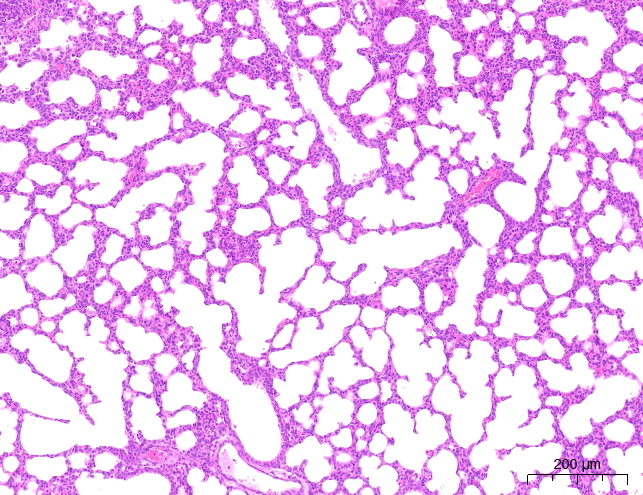

Supplement: Supplementary file 1 [file DataSheet1.zip › data and figures/HE/nc+a.jpg]

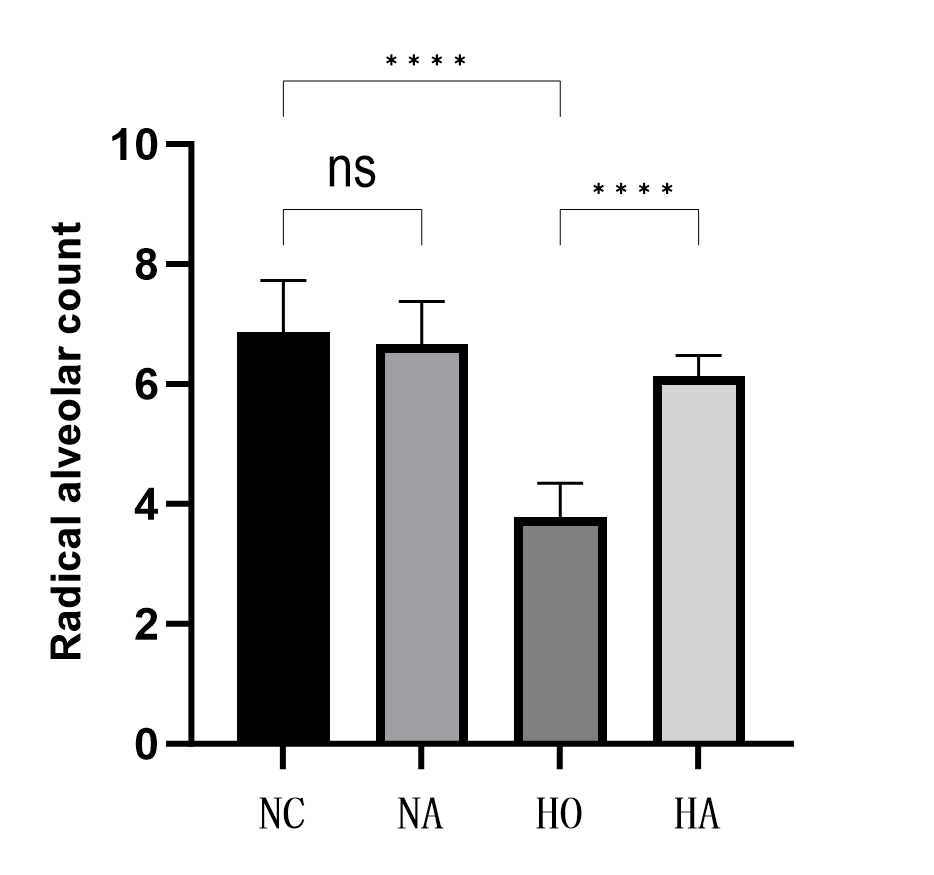

Supplement: Supplementary file 1 [file DataSheet1.zip › data and figures/HE/RAC.tif]

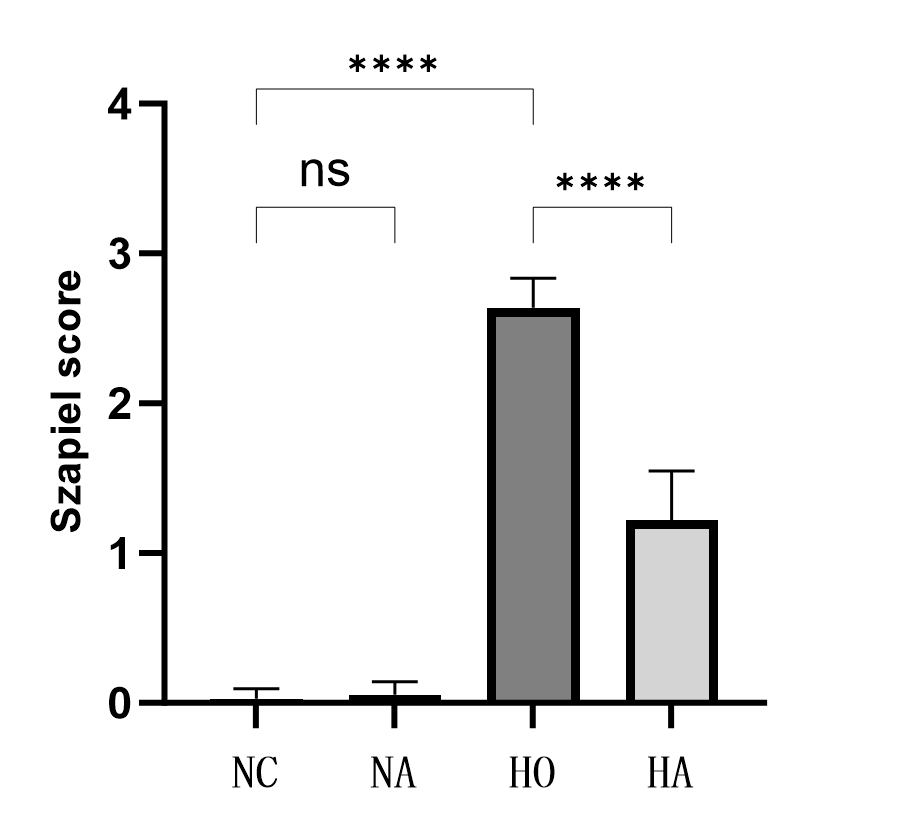

Supplement: Supplementary file 1 [file DataSheet1.zip › data and figures/HE/Szapiel score.tif]

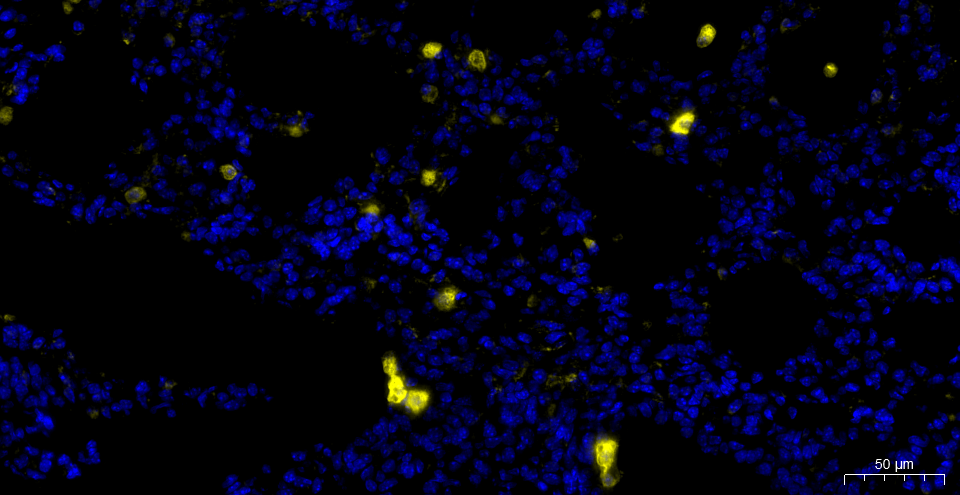

Supplement: Supplementary file 1 [file DataSheet1.zip › data and figures/IF/HA.tif]

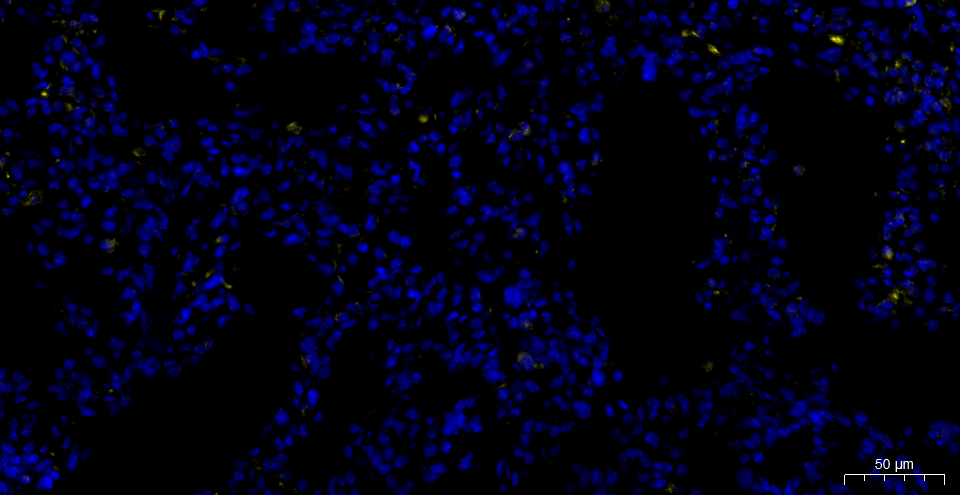

Supplement: Supplementary file 1 [file DataSheet1.zip › data and figures/IF/HO.tif]

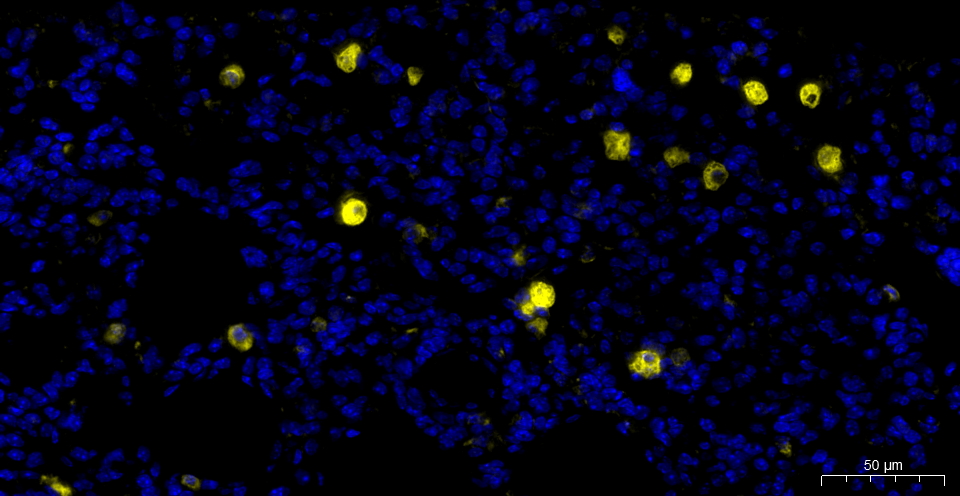

Supplement: Supplementary file 1 [file DataSheet1.zip › data and figures/IF/NA.jpg]

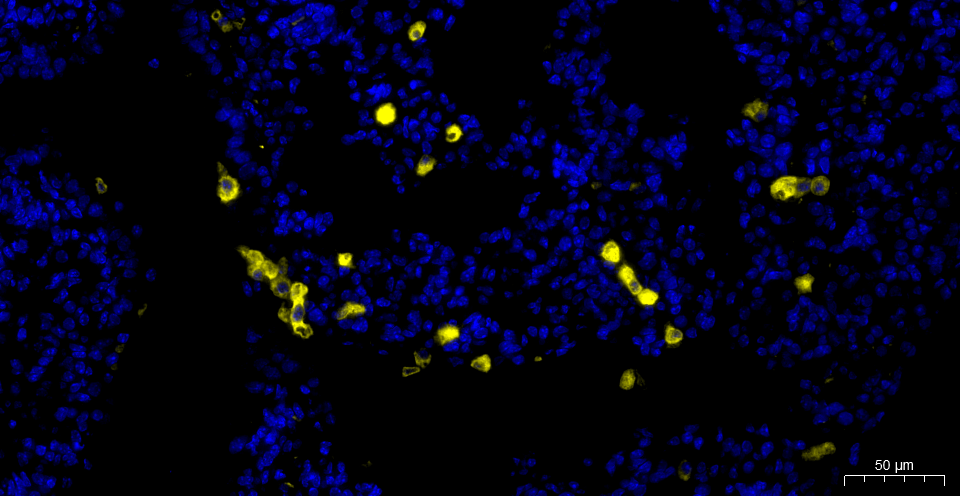

Supplement: Supplementary file 1 [file DataSheet1.zip › data and figures/IF/NC.tif]

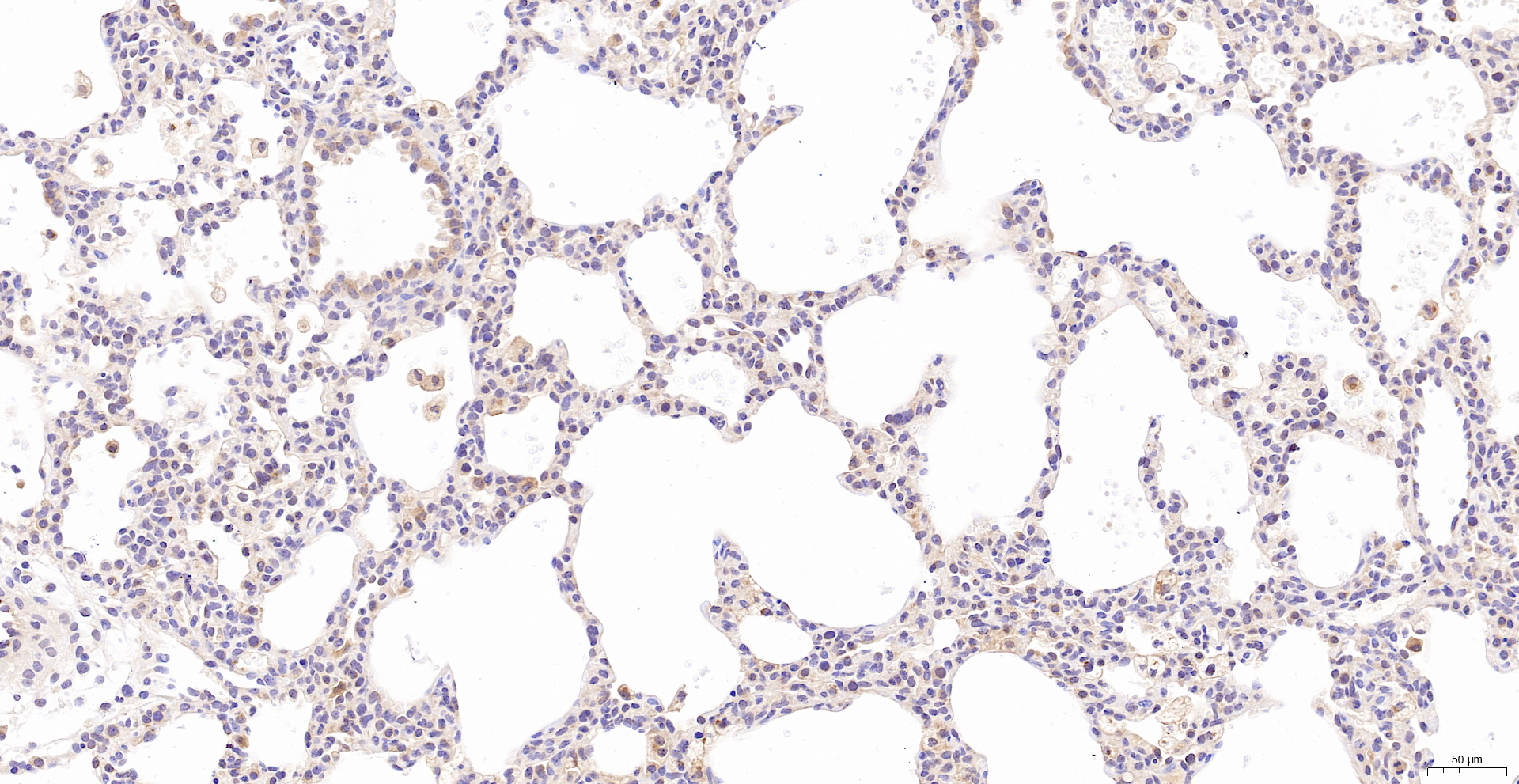

Supplement: Supplementary file 1 [file DataSheet1.zip › data and figures/IHC TGF-a┬/HA.jpg]

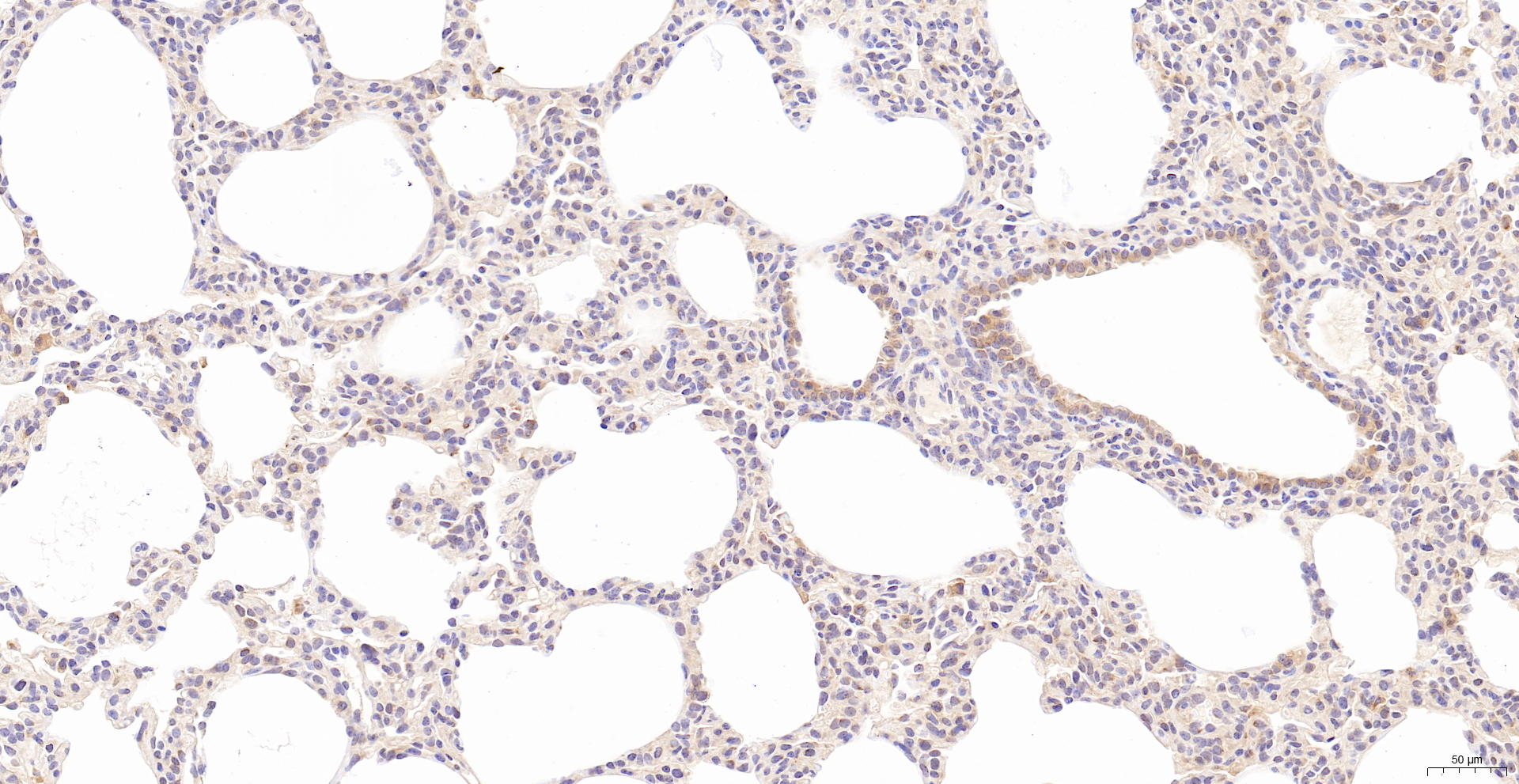

Supplement: Supplementary file 1 [file DataSheet1.zip › data and figures/IHC TGF-a┬/HO.jpg]

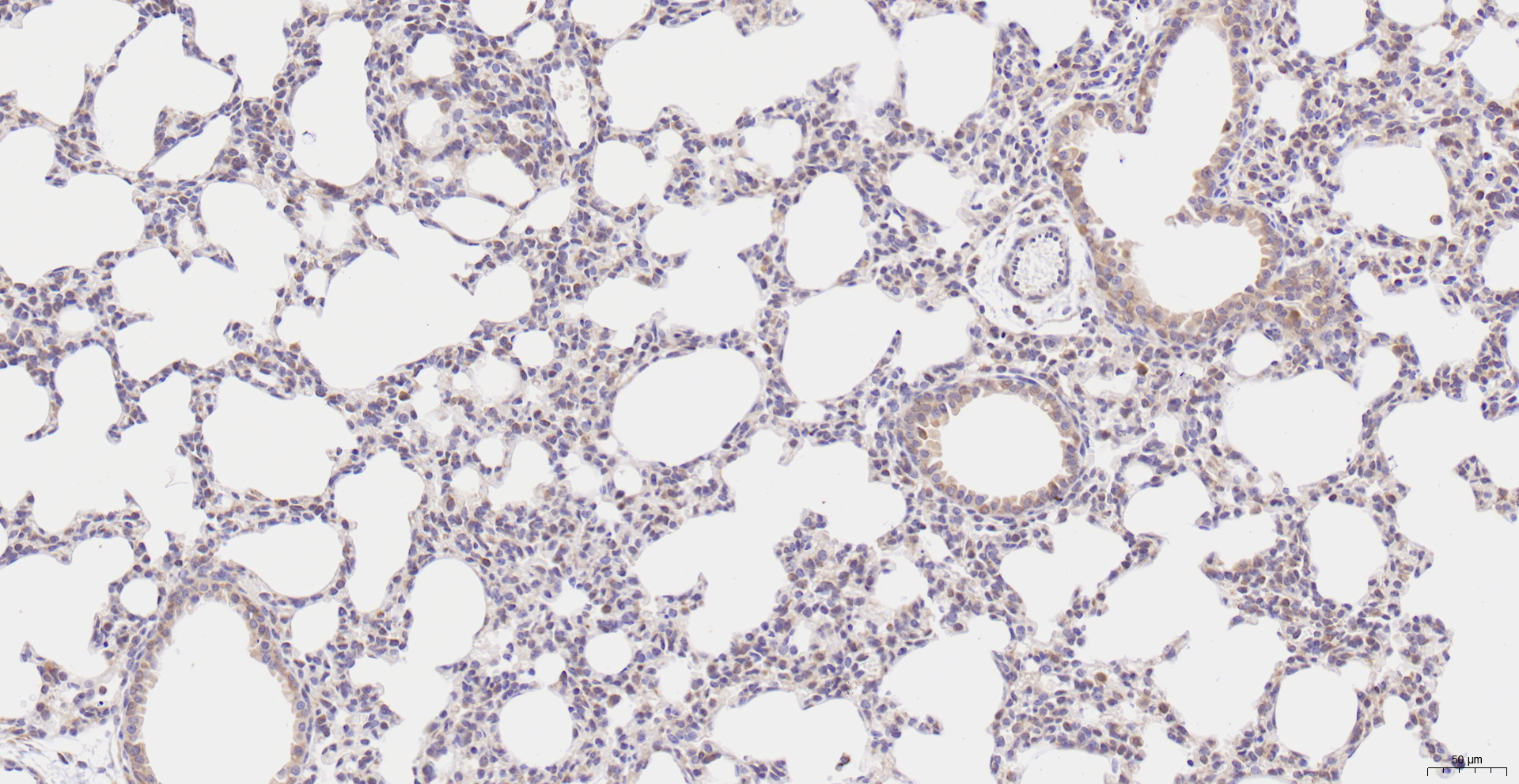

Supplement: Supplementary file 1 [file DataSheet1.zip › data and figures/IHC TGF-a┬/NA.jpg]

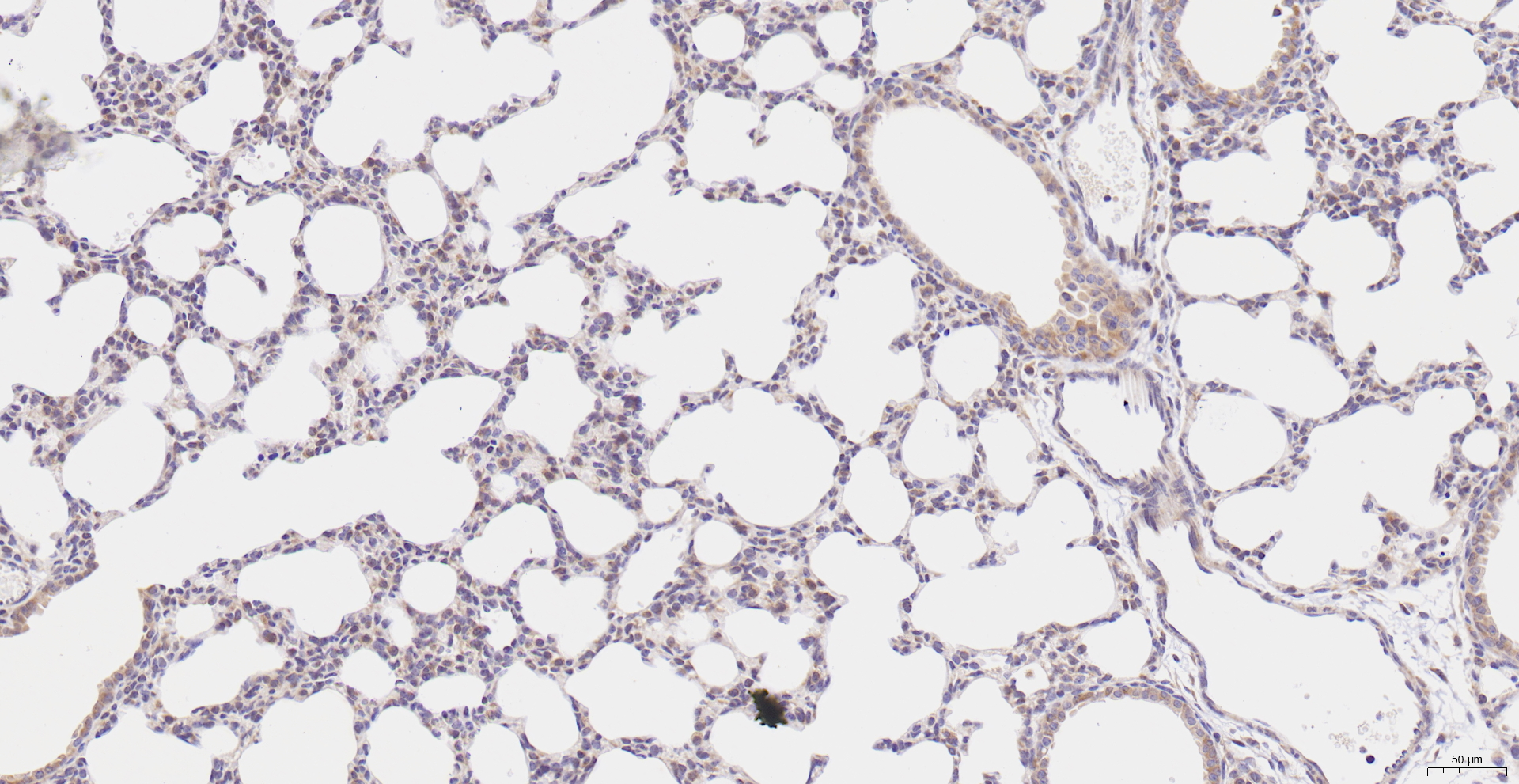

Supplement: Supplementary file 1 [file DataSheet1.zip › data and figures/IHC TGF-a┬/NC.jpg]

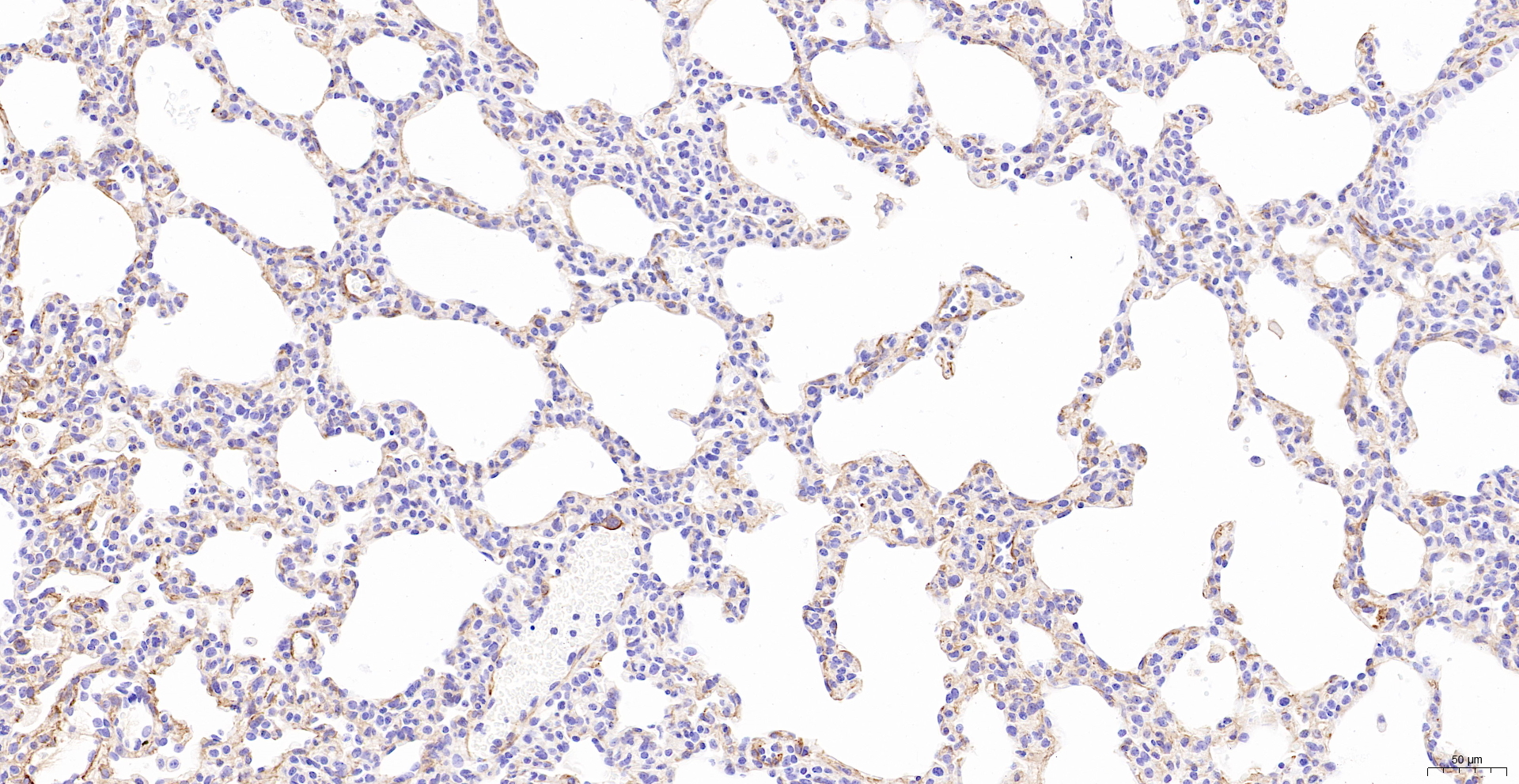

Supplement: Supplementary file 1 [file DataSheet1.zip › data and figures/IHC a┴-SMA/HA.jpg]

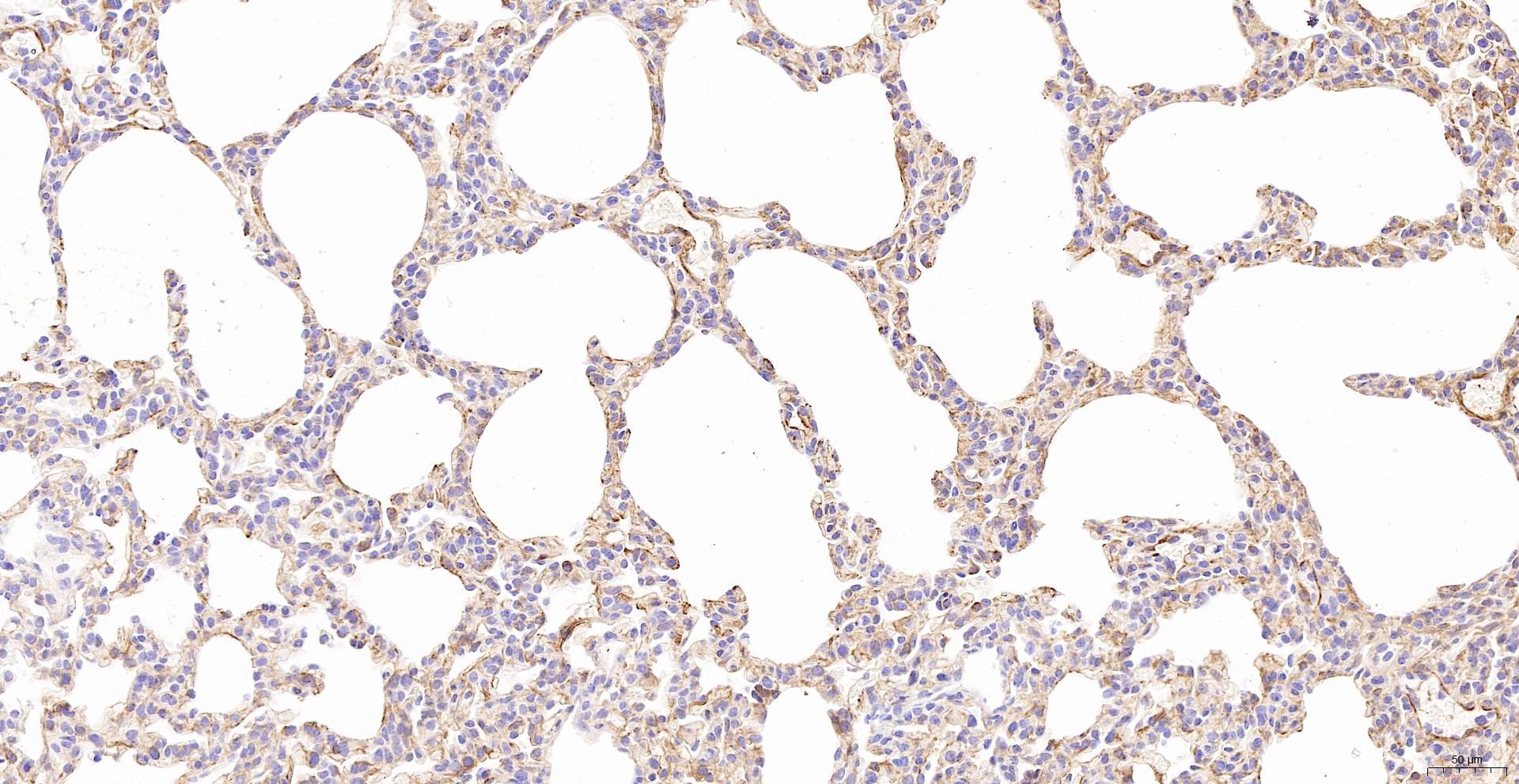

Supplement: Supplementary file 1 [file DataSheet1.zip › data and figures/IHC a┴-SMA/HO.jpg]

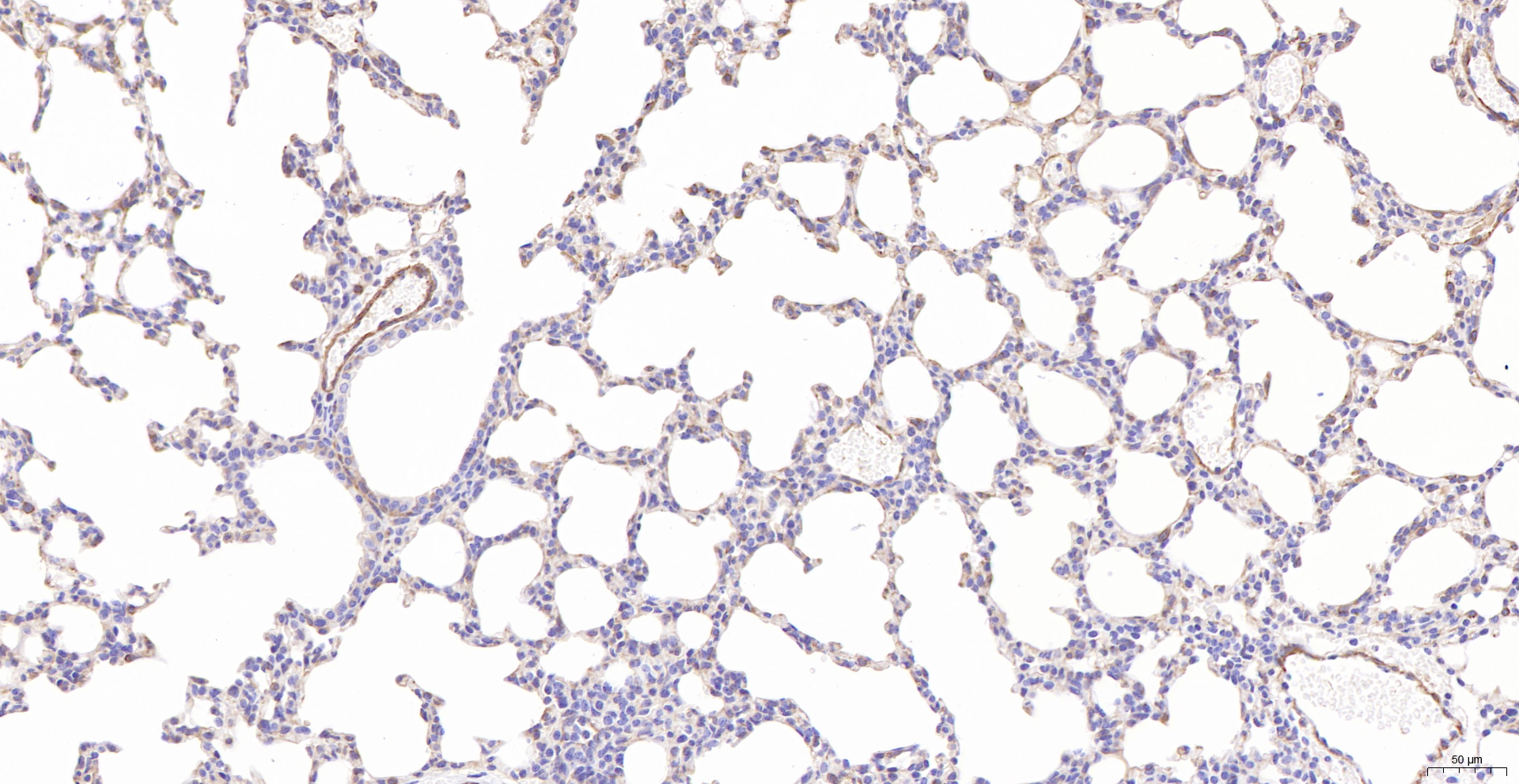

Supplement: Supplementary file 1 [file DataSheet1.zip › data and figures/IHC a┴-SMA/NA.jpg]

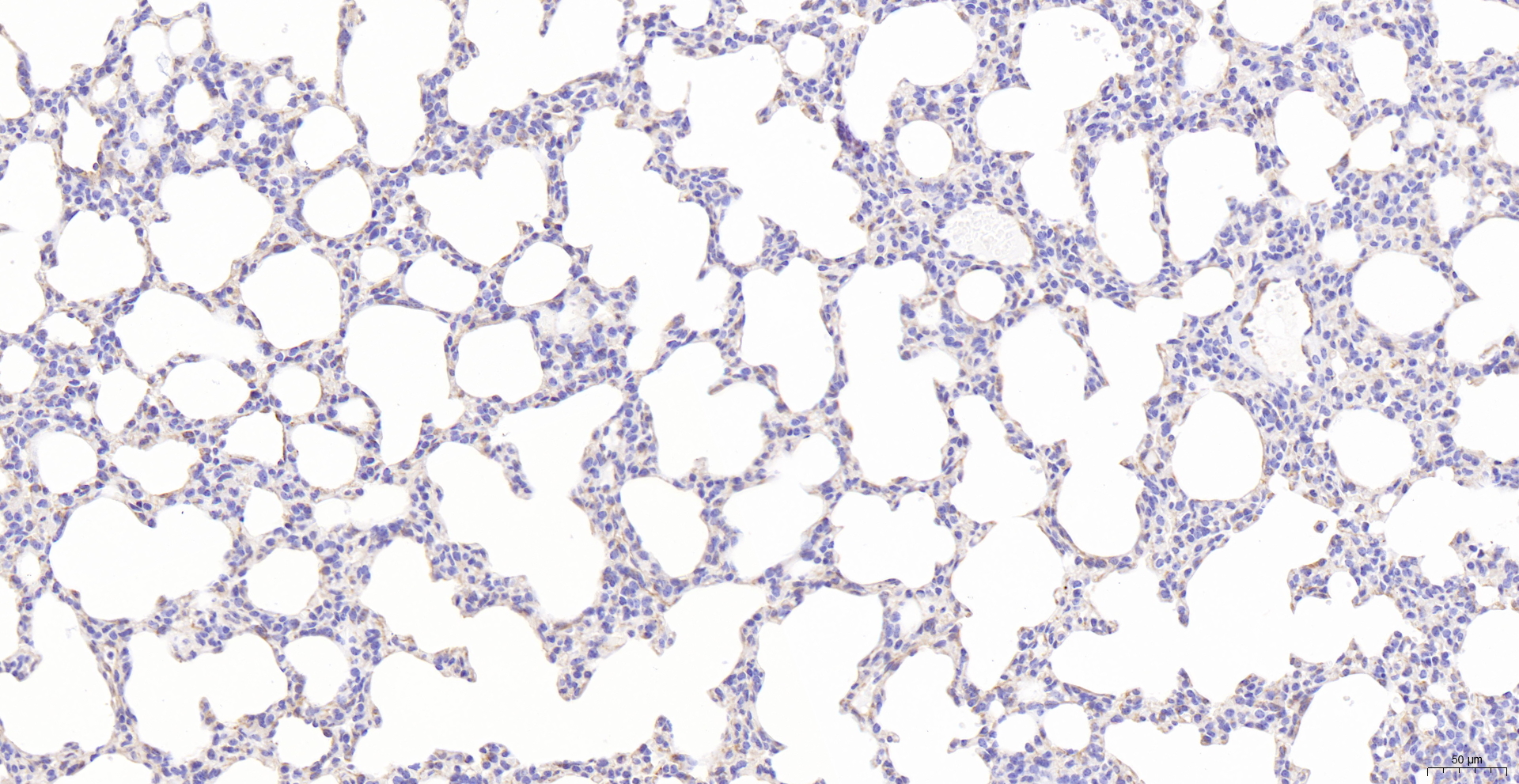

Supplement: Supplementary file 1 [file DataSheet1.zip › data and figures/IHC a┴-SMA/NC.jpg]

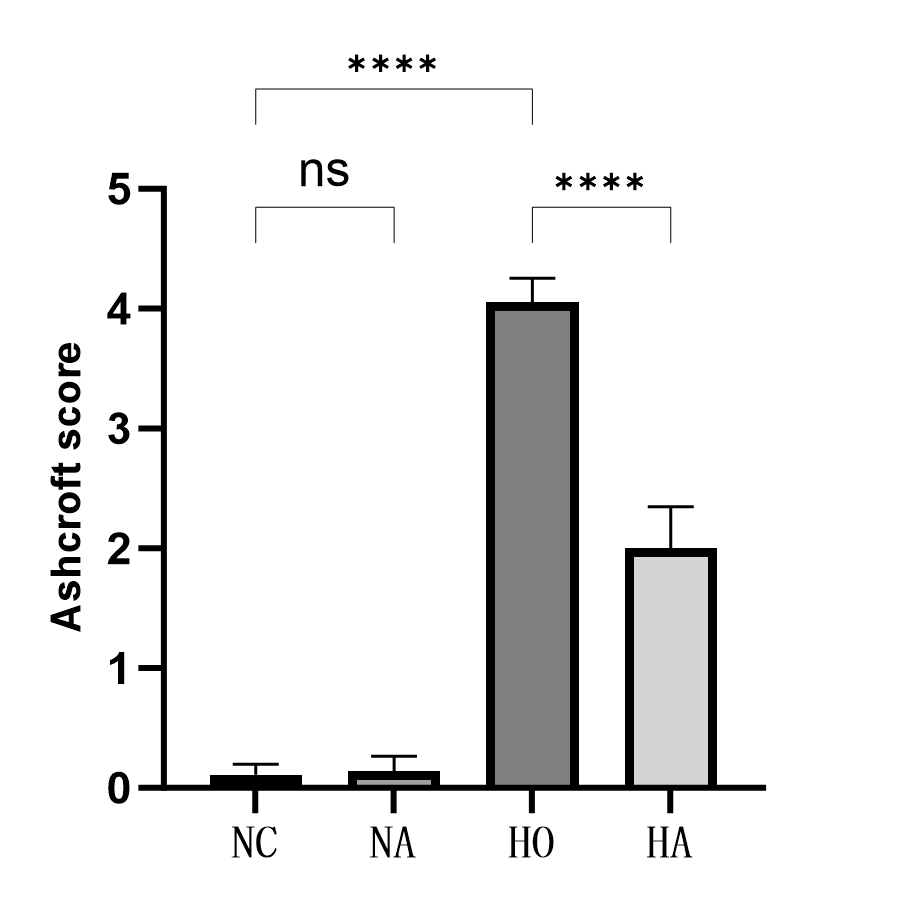

Supplement: Supplementary file 1 [file DataSheet1.zip › data and figures/Masson/Ashcroft score.tif]

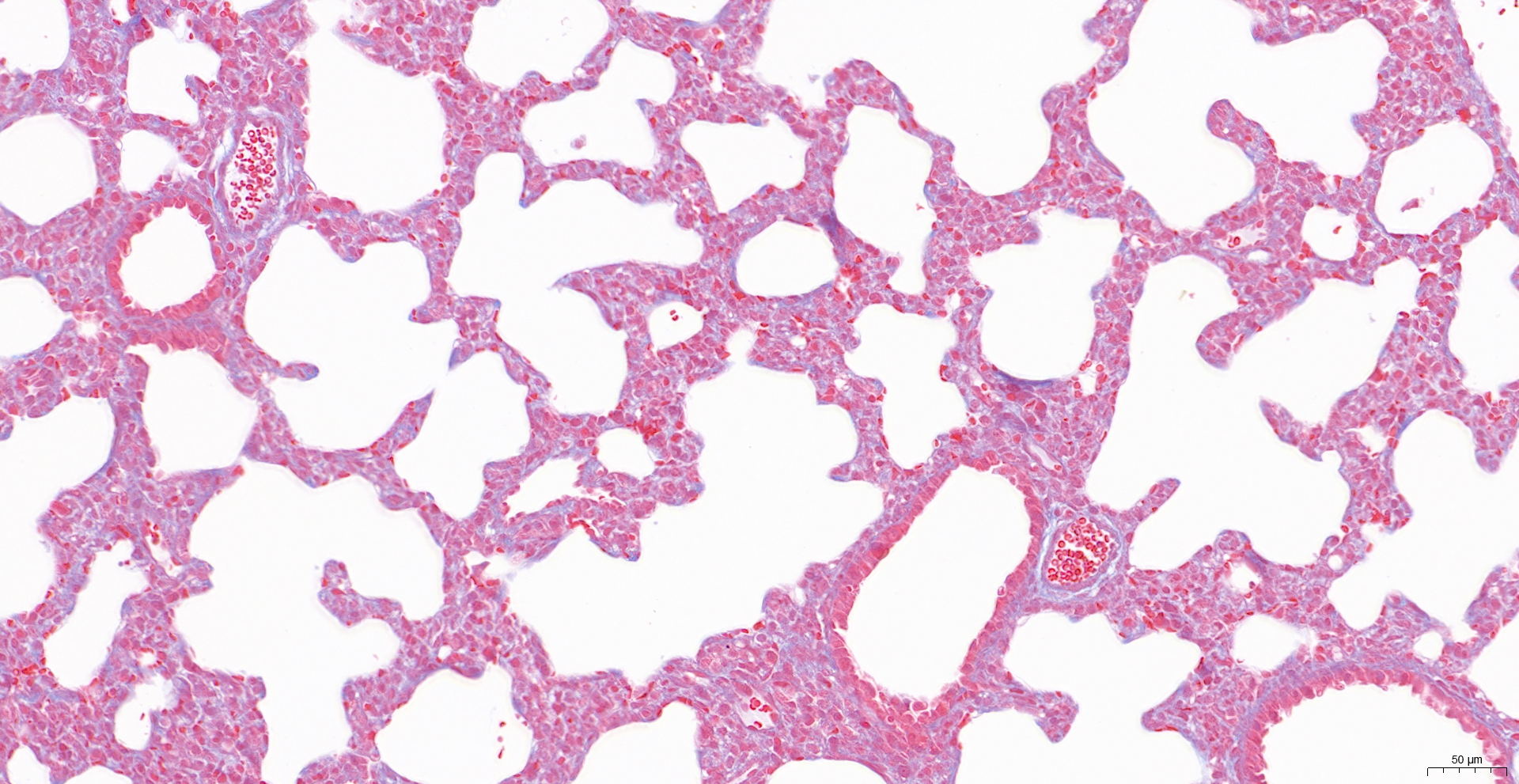

Supplement: Supplementary file 1 [file DataSheet1.zip › data and figures/Masson/HA.jpg]

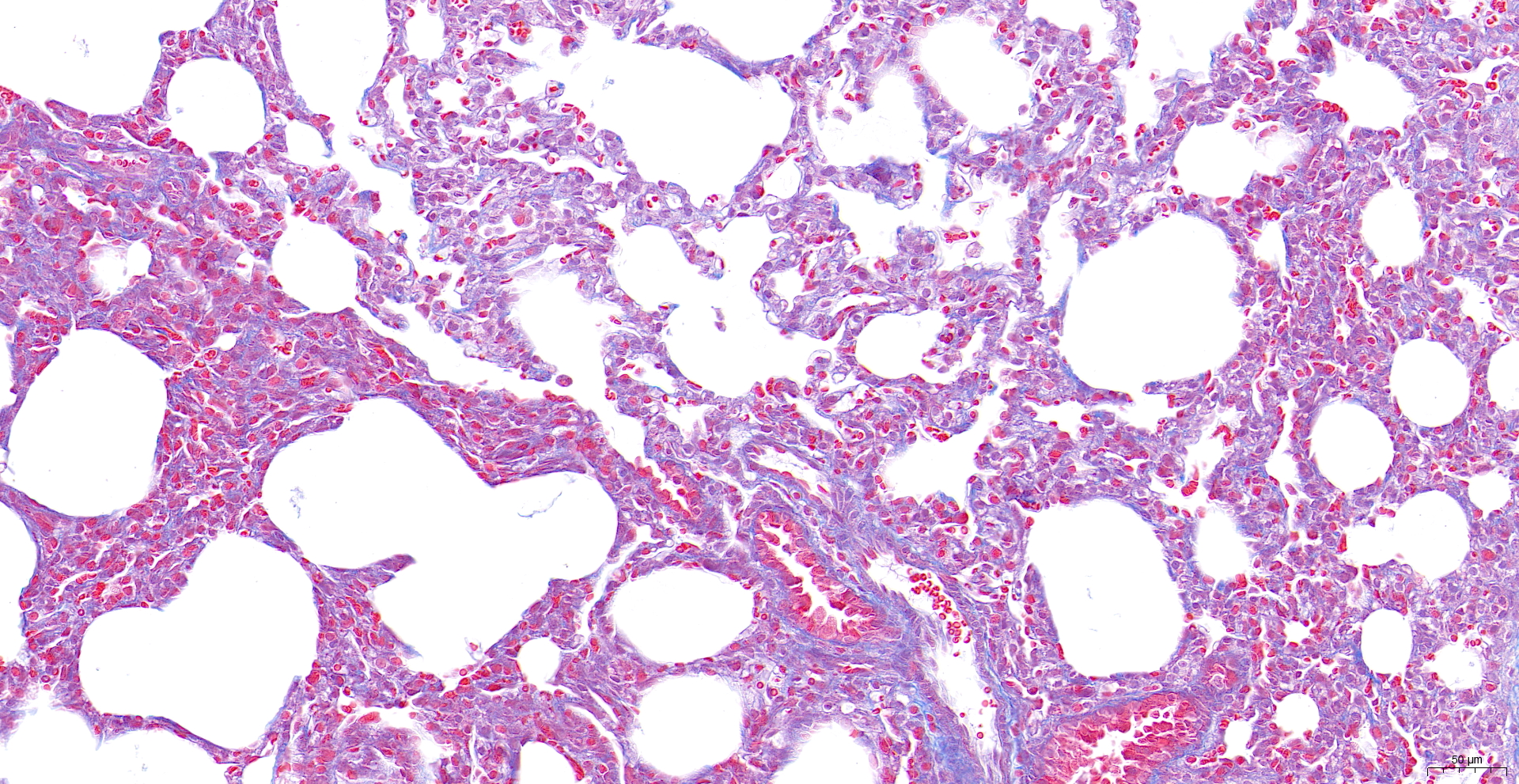

Supplement: Supplementary file 1 [file DataSheet1.zip › data and figures/Masson/HO.jpg]

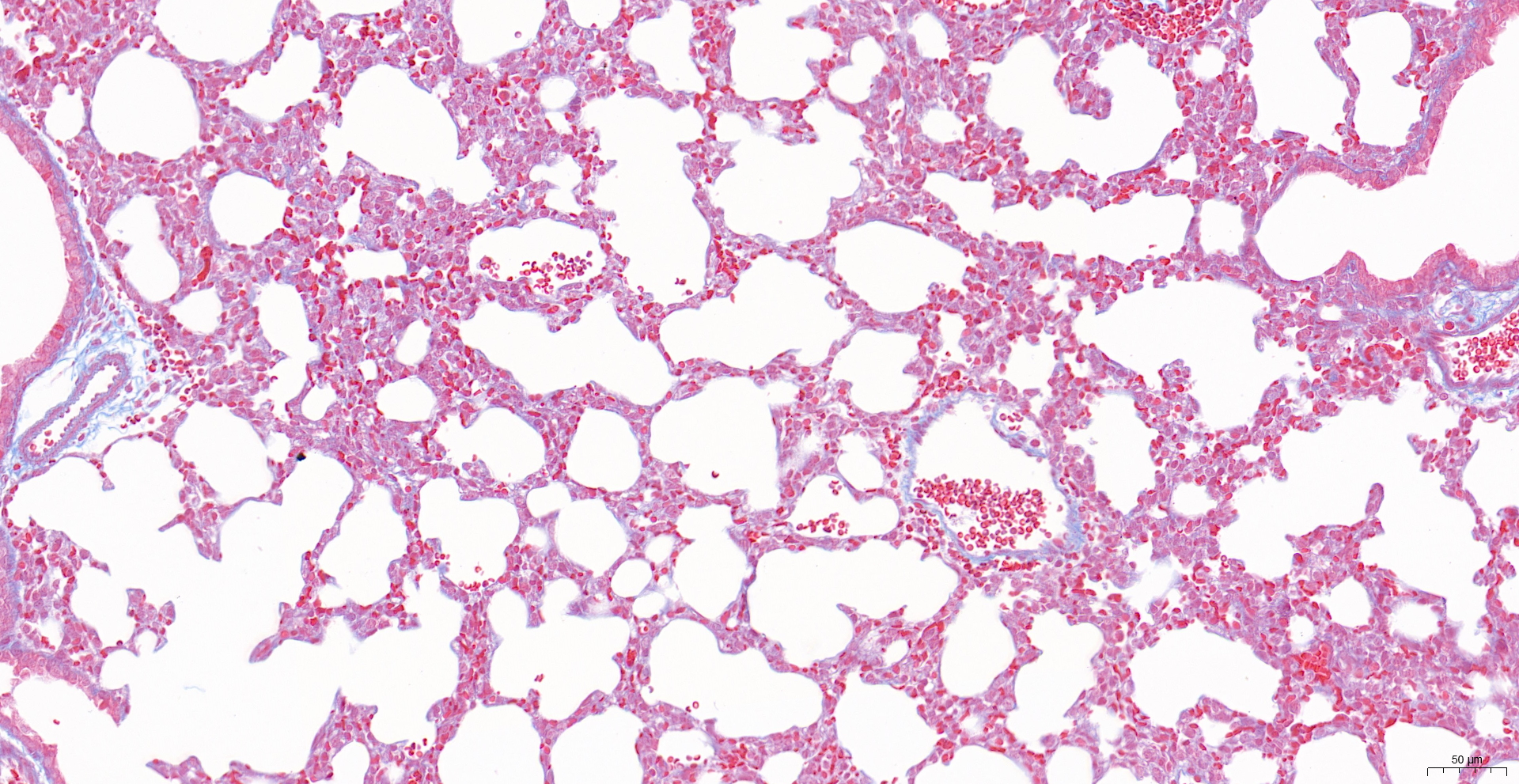

Supplement: Supplementary file 1 [file DataSheet1.zip › data and figures/Masson/NA.jpg]

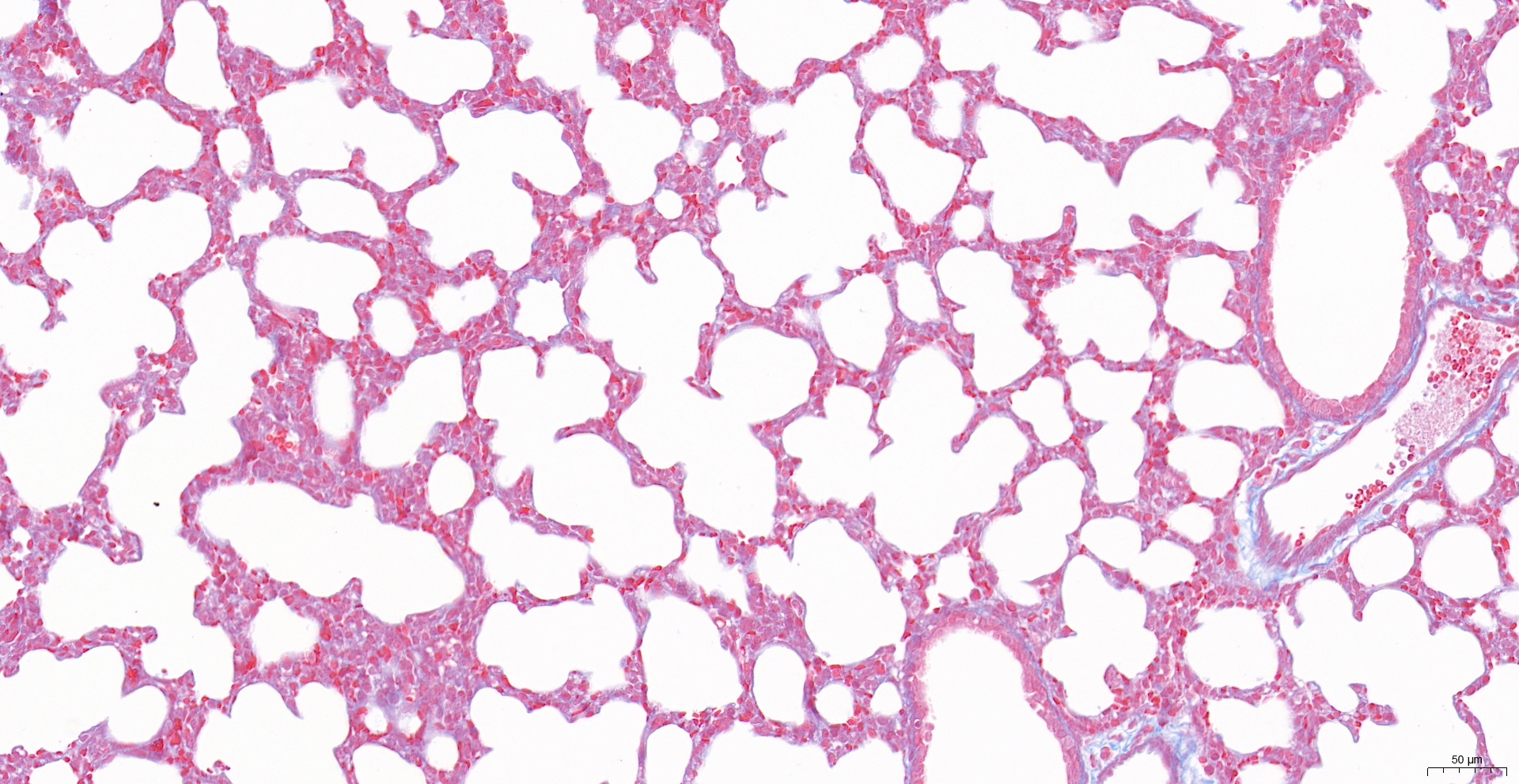

Supplement: Supplementary file 1 [file DataSheet1.zip › data and figures/Masson/NC.jpg]

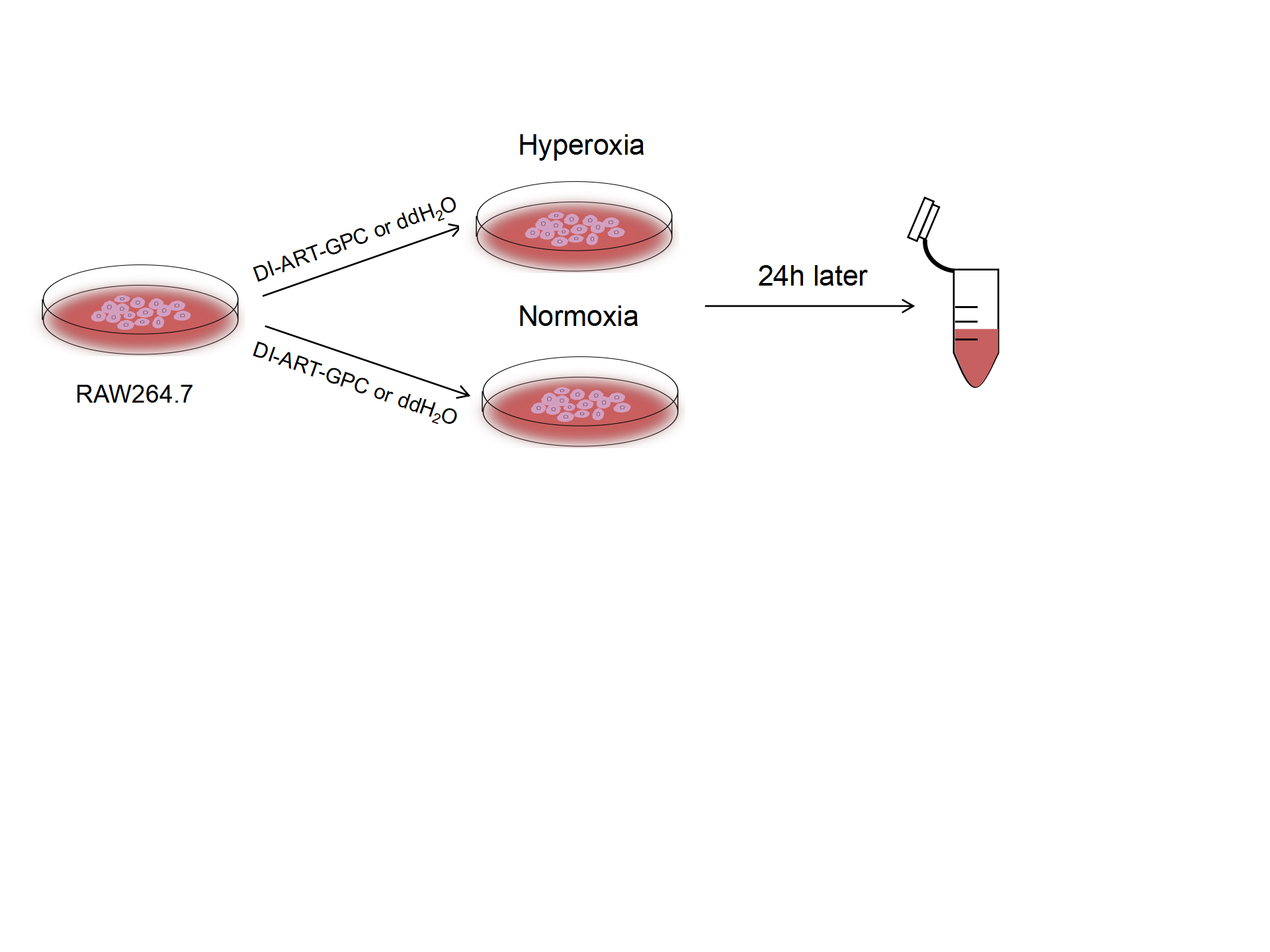

Supplement: Supplementary file 1 [file DataSheet1.zip › data and figures/Molding drawing/in vitro_02.tif]

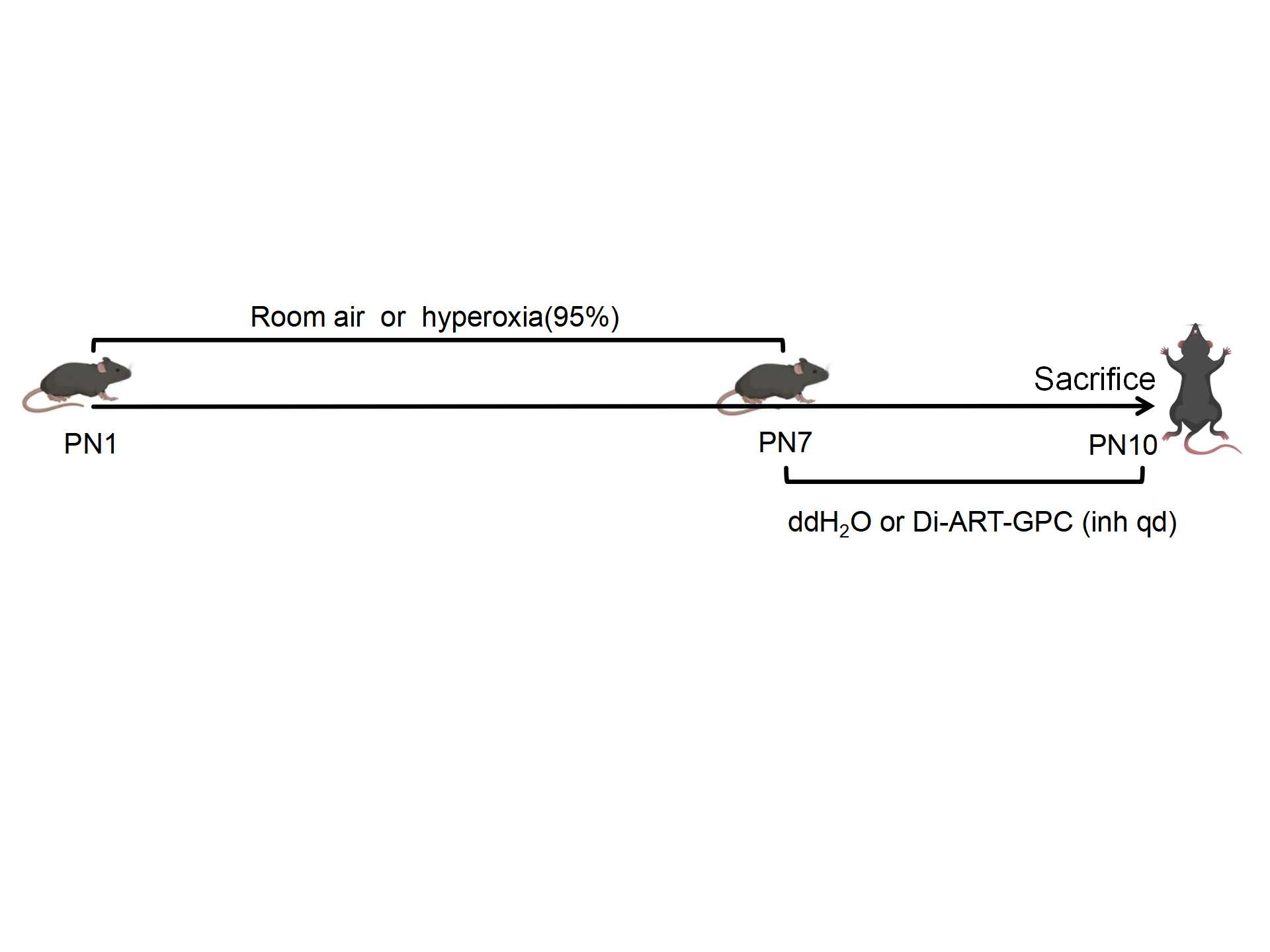

Supplement: Supplementary file 1 [file DataSheet1.zip › data and figures/Molding drawing/in vivo_01.tif]

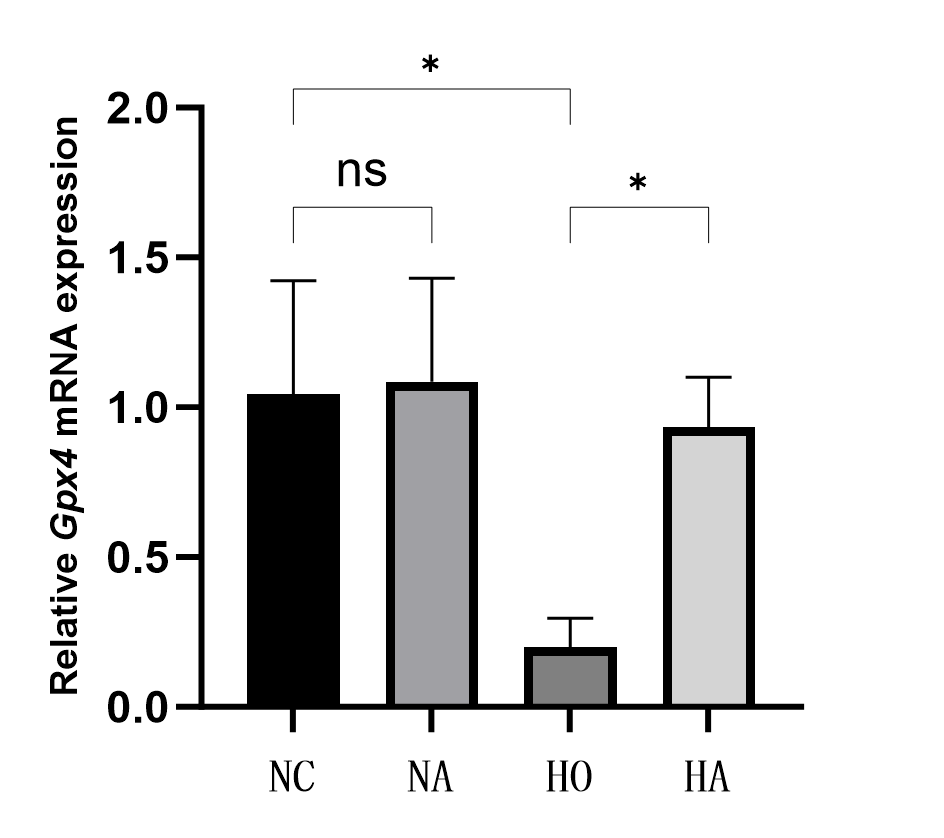

Supplement: Supplementary file 1 [file DataSheet1.zip › data and figures/Rt-qPCR/PCR GPX4 in cell.tif]

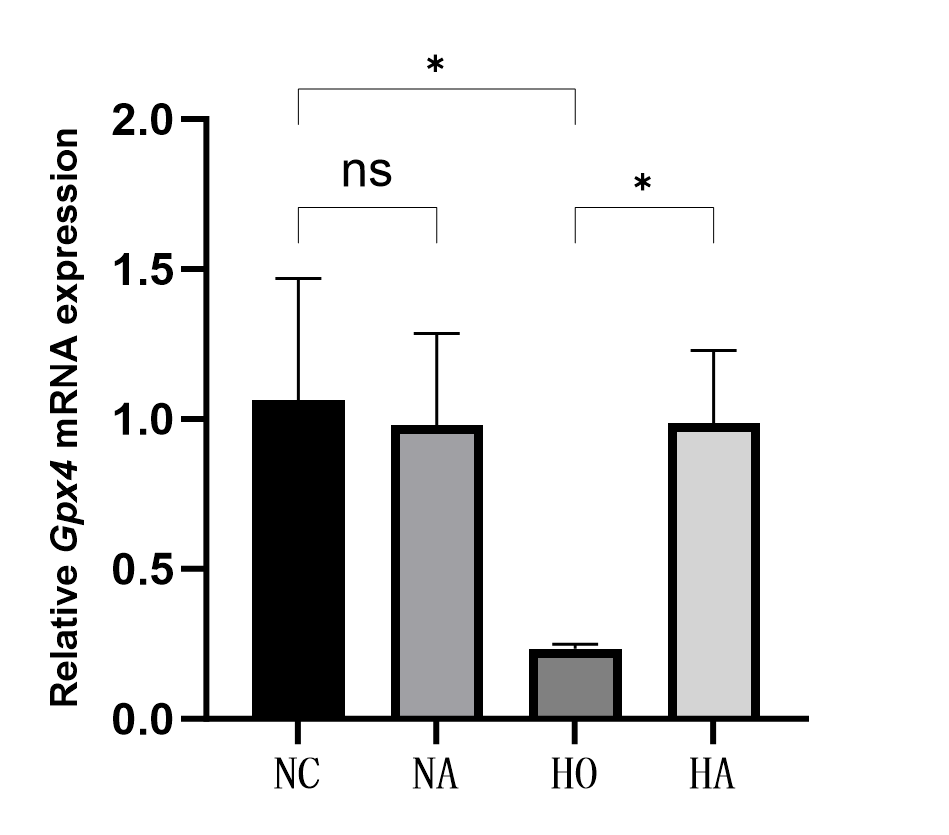

Supplement: Supplementary file 1 [file DataSheet1.zip › data and figures/Rt-qPCR/PCR GPX4 in lung.tif]

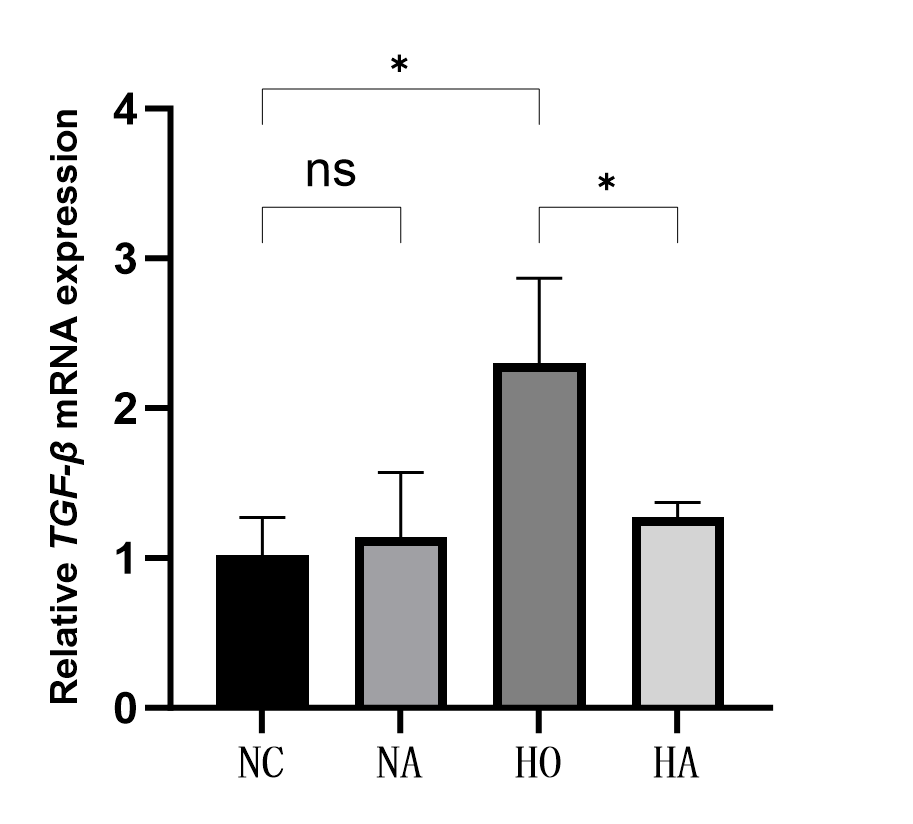

Supplement: Supplementary file 1 [file DataSheet1.zip › data and figures/Rt-qPCR/PCR TGF-a┬ in cell.tif]

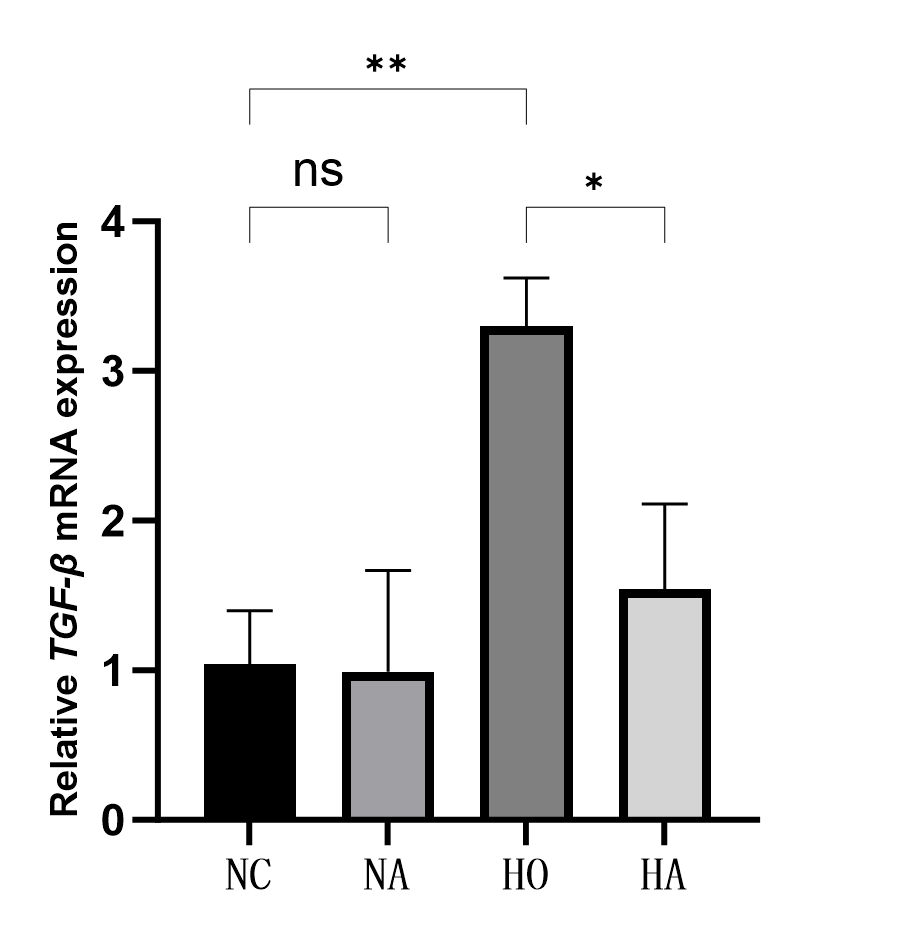

Supplement: Supplementary file 1 [file DataSheet1.zip › data and figures/Rt-qPCR/PCR TGF-a┬ in lung.tif]

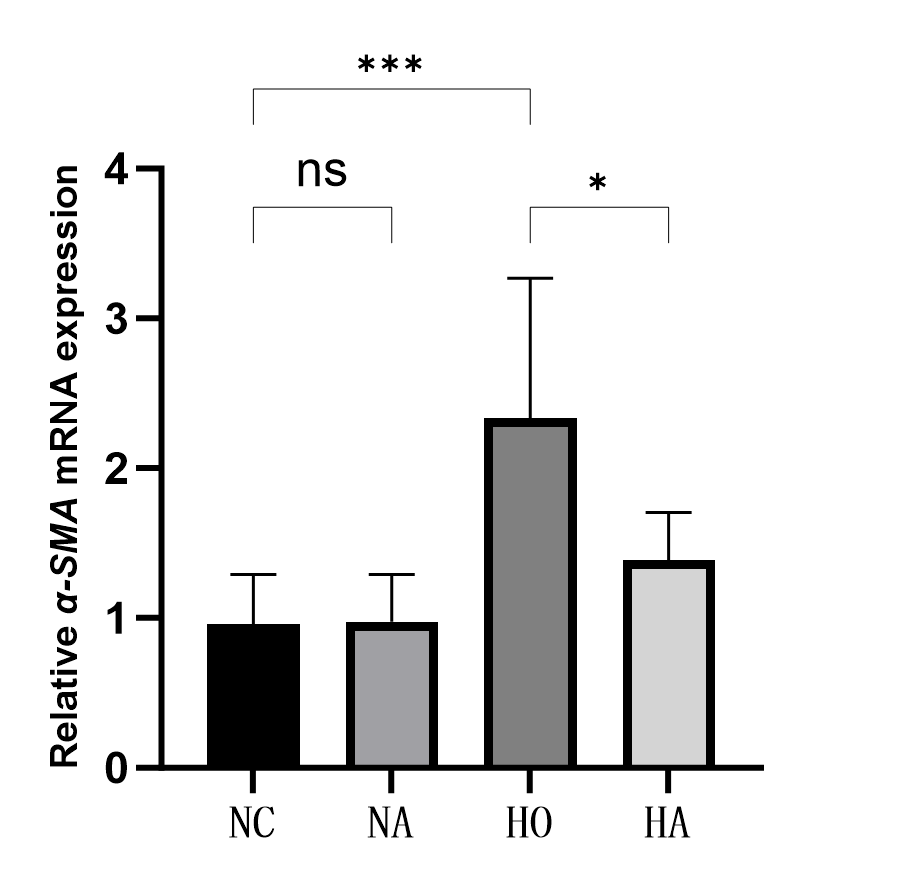

Supplement: Supplementary file 1 [file DataSheet1.zip › data and figures/Rt-qPCR/PCR a┴-SMA in lung.tif]

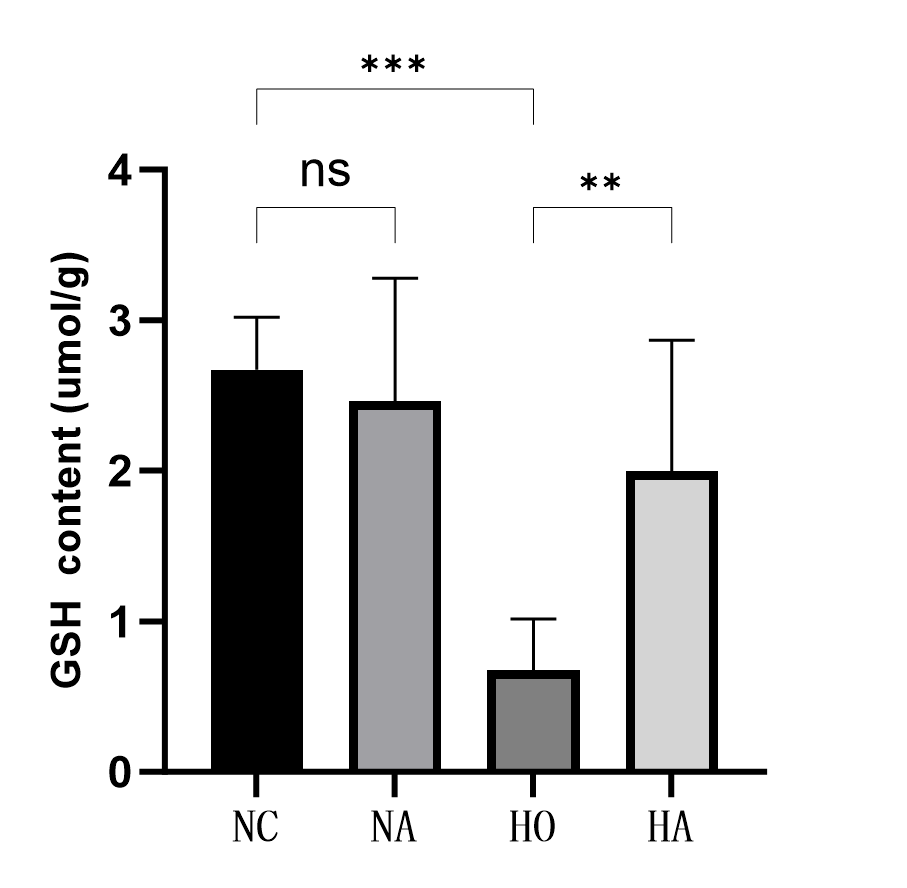

Supplement: Supplementary file 1 [file DataSheet1.zip › data and figures/SOD-MDA-GSH/GSH.tif]

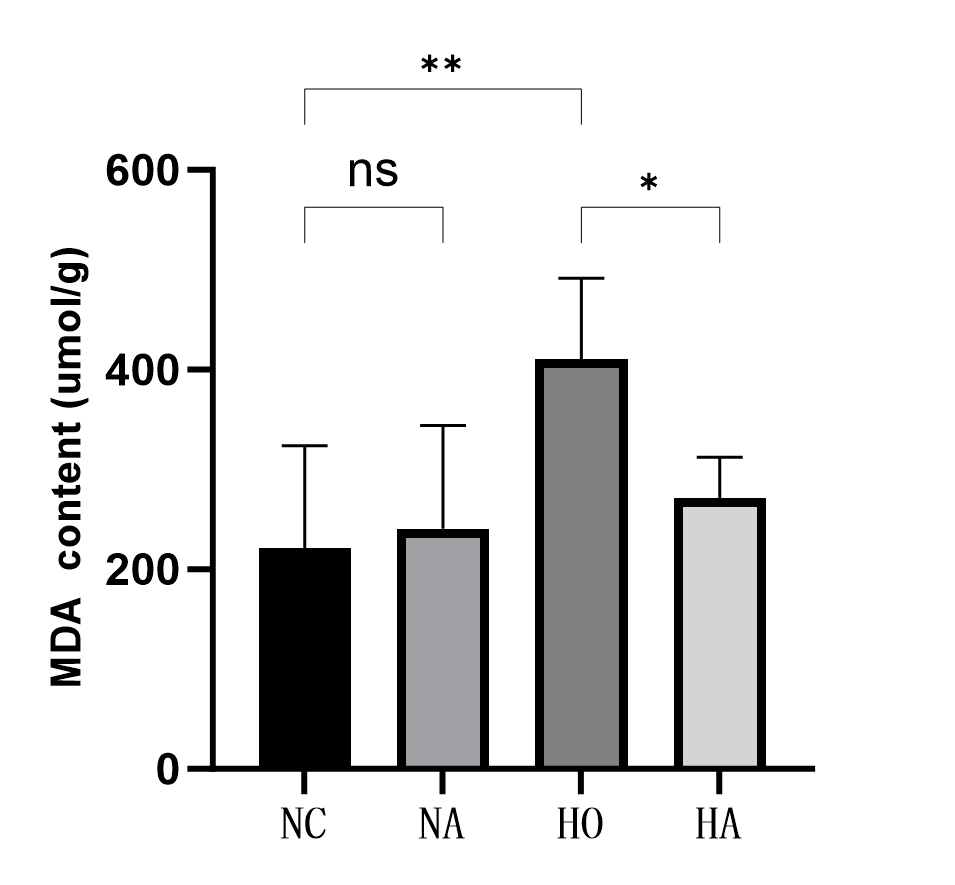

Supplement: Supplementary file 1 [file DataSheet1.zip › data and figures/SOD-MDA-GSH/MDA.tif]

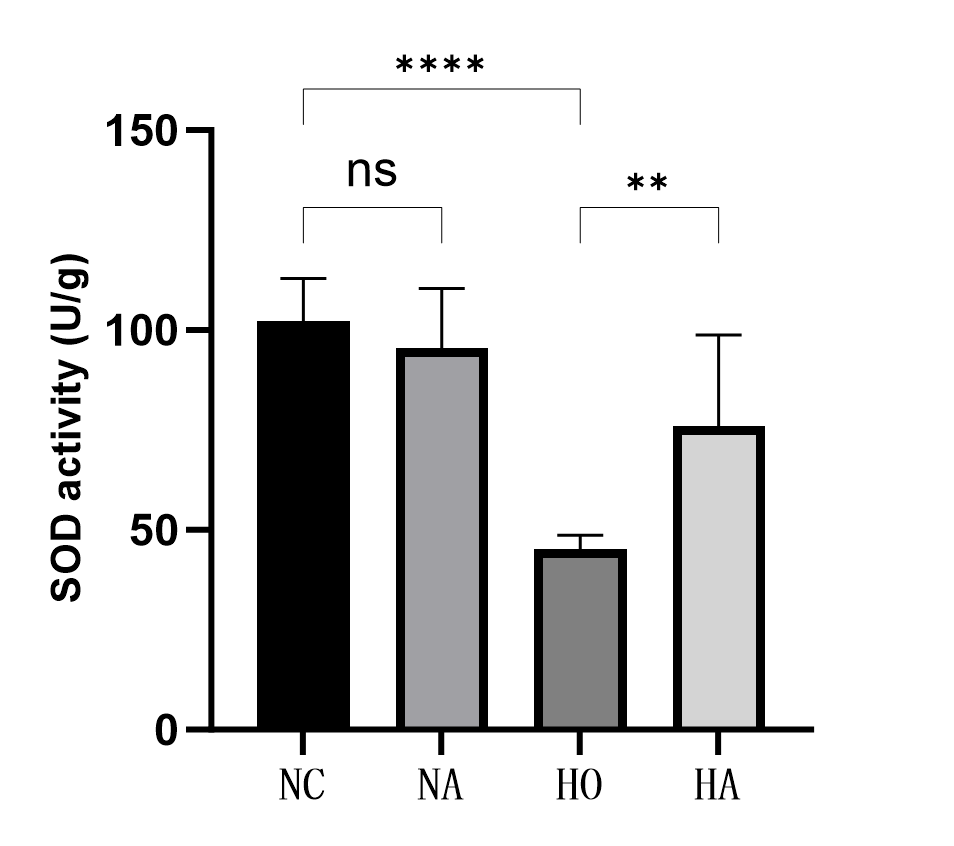

Supplement: Supplementary file 1 [file DataSheet1.zip › data and figures/SOD-MDA-GSH/SOD.tif]

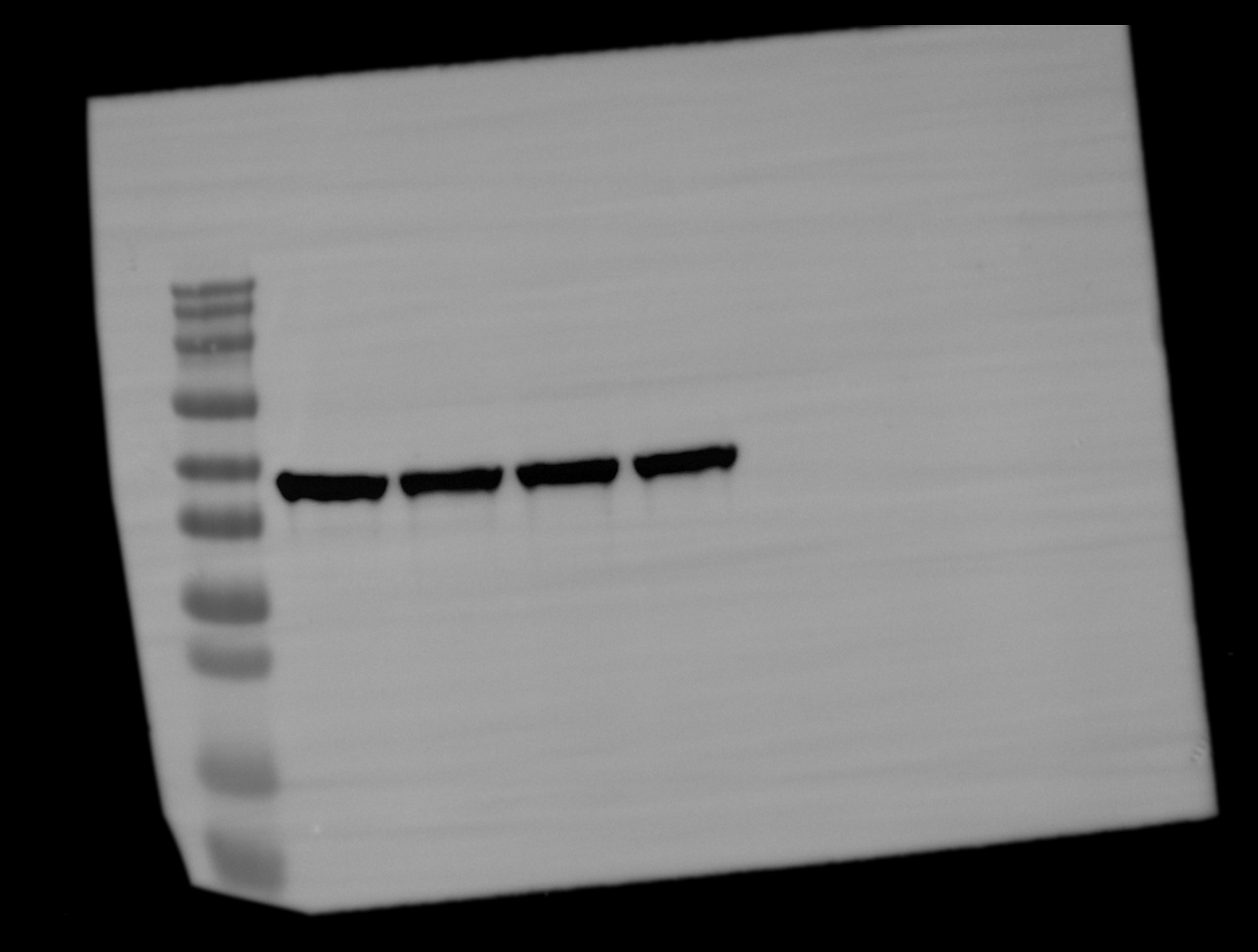

Supplement: Supplementary file 1 [file DataSheet1.zip › data and figures/WB/ACTIN.tiff]

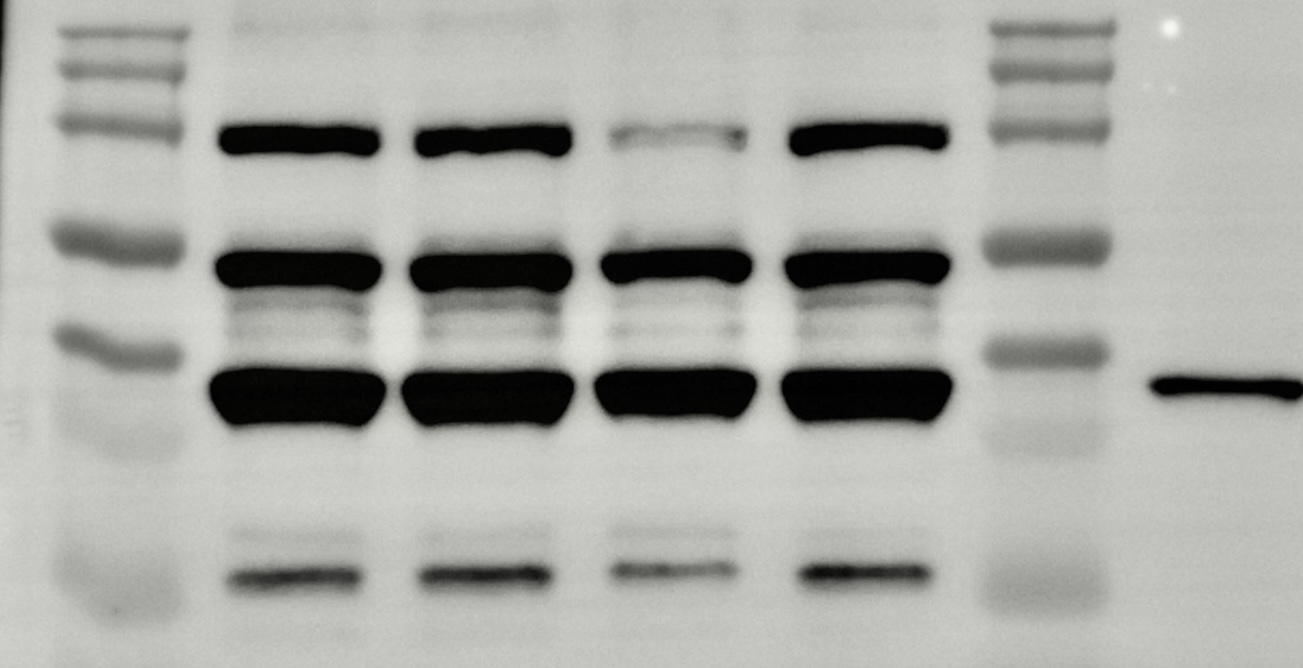

Supplement: Supplementary file 1 [file DataSheet1.zip › data and figures/WB/GPX4 1.jpg]

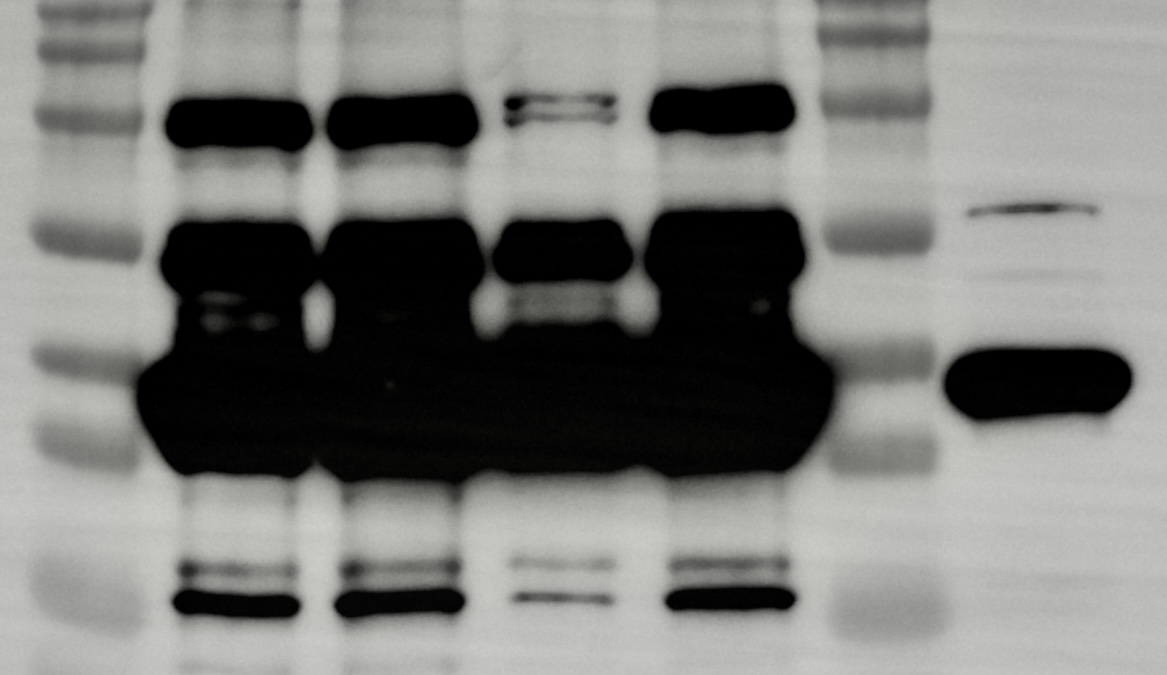

Supplement: Supplementary file 1 [file DataSheet1.zip › data and figures/WB/GPX4 2.jpg]

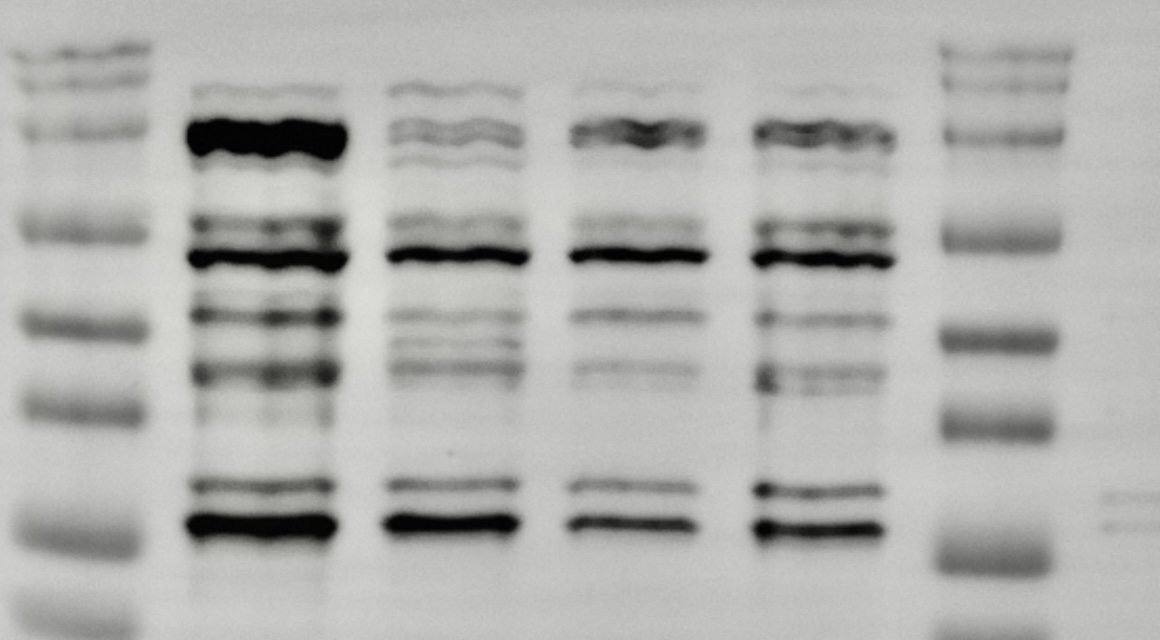

Supplement: Supplementary file 1 [file DataSheet1.zip › data and figures/WB/GPX4 3.jpg]

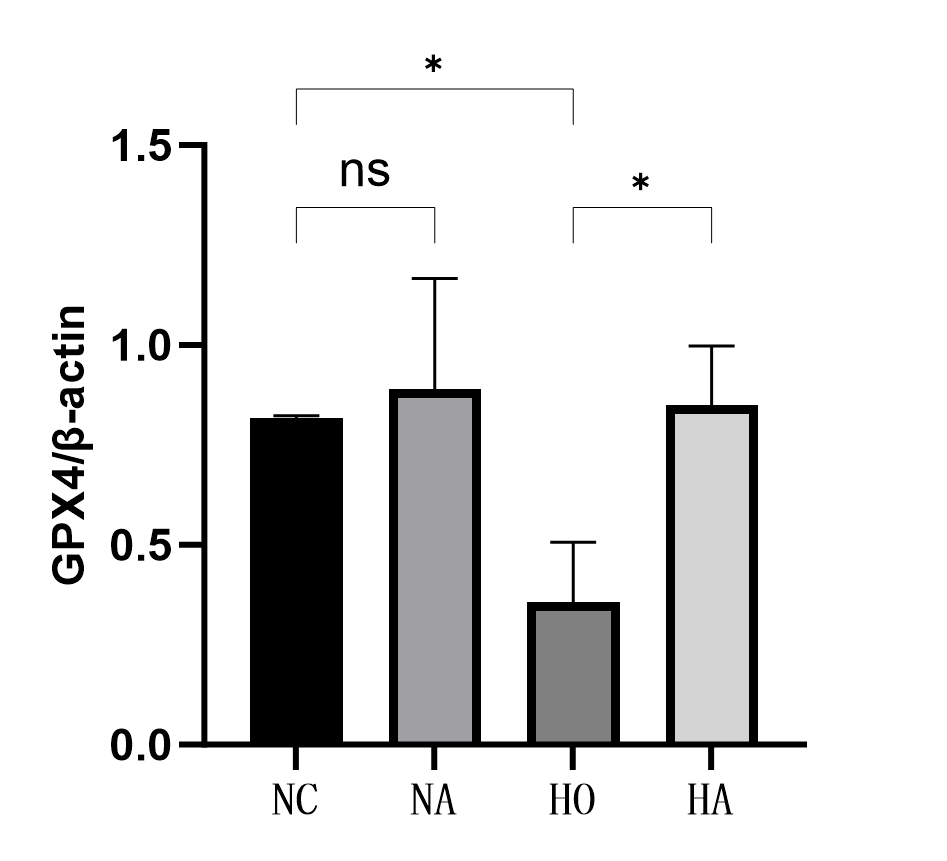

Supplement: Supplementary file 1 [file DataSheet1.zip › data and figures/WB/gpx4_a┬-actin.tif]
